# Waterborne pathogens detection technologies: advances, challenges, and future perspectives

**DOI:** 10.3389/fmicb.2023.1286923

**Published:** 2023-11-23

**Authors:** Yoong-Ling Oon, Yoong-Sin Oon, Muhammad Ayaz, Min Deng, Lu Li, Kang Song

**Affiliations:** ^1^Southern Marine Science and Engineering Guangdong Laboratory (Guangzhou), Guangzhou, China; ^2^State Key Laboratory of Freshwater Ecology and Biotechnology, Institute of Hydrobiology, Chinese Academy of Sciences, Wuhan, Hubei, China; ^3^Department of Environmental and Conservation Sciences, University of Swat, Swat, Pakistan; ^4^University of Chinese Academy of Sciences, Beijing, China

**Keywords:** pathogen detection technique, PCR, biosensor, water microbiology, nucleic acid amplification, LAMP, NGS, artificial intelligence

## Abstract

The World Health Organization (WHO) estimated that pathogens like Escherichia coli, primarily linked to food and water contamination, are associated with 485,000 deaths from diarrheal diseases annually, translating to a staggering worldwide economic loss of nearly 12 billion USD per annum. International organizations like the WHO and United Nations Children’s Fund (UNICEF) have established related guidelines and criteria for pathogenic detection technologies and driving the search for innovative and efficient detection methods. This comprehensive review examines the trajectory of waterborne pathogenic bacteria detection technologies from traditional techniques, i.e., culture-based methods, to current detection methods including various forms of polymerase chain reaction (PCR) techniques [qualitative real-time PCR, digital PCR, ELISA, loop-mediated isothermal amplification, next-generation sequencing (NGS)] and to emerging techniques, i.e., biosensors and artificial intelligence (AI). The scope of the review paper focuses on waterborne pathogenic bacteria that are recognized as human pathogens, posing tangible threats to public health through waterborne. The detection techniques’ merits, constraints, research gaps and future perspectives are critically discussed. Advancements in digital droplet PCR, NGS and biosensors have significantly improved sensitivity and specificity, revolutionizing pathogen detection. Additionally, the integration of artificial intelligence (AI) with these technologies has enhanced detection accuracy, enabling real-time analysis of large datasets. Molecular-based methods and biosensors show promise for efficient water quality monitoring, especially in resource-constrained settings, but on-site practical implementation remains a challenge. The pairwise comparison metrics used in this review also offer valuable insights into quick evaluation on the advantages, limitations and research gaps of various techniques, focusing on their applicability in field settings and timely analyses. Future research efforts should focus on developing robust, cost-effective and user-friendly techniques for routine waterborne bacteria monitoring, ultimately safeguarding global water supplies and public health, with AI and data analysis playing a crucial role in advancing these methods for a safer environment.

## Graphical Abstract

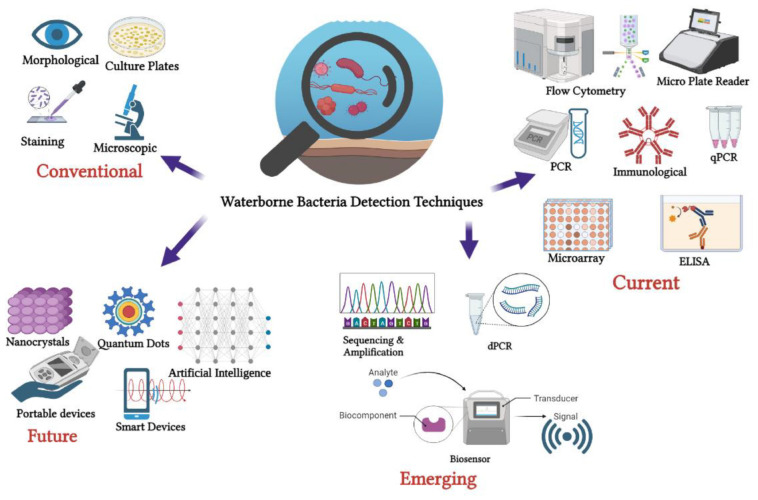

## 1 Introduction

Water is one of the most essential natural resources for the continuation of life on earth and the quality of water quality is a critical factor for the survival and wellbeing of all living organisms. Human activity depends heavily on water sources around the world in terms of economic development, social welfare, and, most importantly, the provision of food and healthcare. Due to growth and development, water sources are more susceptible to various contaminants, including biological and non-biological. There is worldwide concern regarding the presence of biological contaminants, including waterborne pathogens (Ramírez-Castillo et al., 2015; Kumar et al., 2019), where they have been detected in various types of water sources including freshwater such as rivers and lakes (Cui et al., 2019), marine water, domestic water (Duressa et al., 2019) etc. Outbreaks of waterborne epidemics have occurred in various countries, primarily attributed to pathogenic bacteria (Ambili and Sebastian, 2021; Paruch, 2022), viruses and protozoa (Artika et al., 2020).

The recent lesson from the Coronavirus disease 2019 (COVID-19) pandemic not only raises awareness of the potential zoonotic and emerging infectious diseases but also prompts us of the ongoing impact of the risk of waterborne diseases. Waterborne diseases are a major public health concern since they result in significant morbidity and mortality worldwide. Over seven million people in the United States are affected annually by waterborne diseases, underscoring a substantial economic burden of 2.2–3.7 billion USD, including medical costs, lost productivity and mortality (DeFlorio-Barker et al., 2018). The World Health Organization (WHO) estimated that 485,000 deaths each year were associated with diarrheal diseases, primarily linked to food and water contaminated with pathogens like *Escherichia coli* (*E. coli*) (World Health Organization [WHO], 2023). This translates into a staggering economic loss of nearly 12 billion USD per annum worldwide (Alhamlan et al., 2015).

To mitigate this public health crisis, various international bodies such as WHO, the United States Environmental Protection Agency (US EPA), the European Union as well as other respective countries, have delineated specific acceptable limits of pathogens in water. Monitoring these limits necessitates a meticulous examination of indicator bacteria such as coliforms, *E. coli* and *P. aeruginosa*. Notably, *E. coli* is the WHO’s preferred marker for fecal contamination of drinking water (World Health Organization [WHO], 2017), while *Enterococci* serves as indicator bacteria for evaluating the quality of bathing water (European Union [EU], 2006).

The challenge to ensure microbiological water safety becomes more profound in resource-constrained settings, particularly in low-income countries. The Sustainable Development Goals (SDGs) aim to overcome this hurdle by tracking global progress in drinking water safety, including *E. coli* measurement in drinking water sources. In recognizing the shortcomings of waterborne pathogen detection method and their application in resource-constrained settings. United Nations Children’s Fund (UNICEF) along with WHO, also introduced a Target Product Profile (TPP) in 2016, incentivizing the development of innovative products capable of detecting low levels of viable *E. coli* in field water samples within 6 h (UNICEF TPP, 2019). In the most recent UNICEF TPP Rapid Water Quality Tests, the updated requirements now aim for a time to result of less than 2 h with a sample of 30 colony-forming units (CFU) (UNICEF, 2023). These changes reflect the ongoing efforts to enhance the efficiency and accuracy of water quality testing methods, making them more suitable for real-time monitoring and ensuring safe water supplies for communities.

Over the years, the quest for such tests has led to the emergence of several alternatives to culture-based standard membrane filtration assays, specifically for detecting *E. coli* and other fecal indicator bacteria. Meeting this demand could promote simple, rapid, scalable and cost-effective microbial water safety tests, particularly benefiting basic settings and non-experts. Culture-based has been regarded as the gold standard for waterborne pathogen detection methods. Nevertheless, these techniques are often constrained by long incubation periods. As technology advances, molecular-based techniques allow for the detection of specific DNA/RNA sequences in target pathogens without the need for culturing, shorter result turnaround time, improved sensitivity, specificity and reproducibility compared to traditional culture-dependent microbiological methods. The drawbacks are complex processes require specialized training, which deters on-site water testing (Paruch, 2022).

Molecular detection techniques have evolved from conventional PCR to real-time quantitative PCR (qPCR) (Saleem et al., 2023), DNA microarrays (Inoue et al., 2015), enzyme-linked immunosorbent assay (ELISA), digital droplet PCR (ddPCR) (Mauvisseau et al., 2019; Falzone et al., 2020), loop-mediated isothermal amplification (LAMP) (Fu et al., 2021) and high-throughput next-generation DNA sequencing (Garner et al., 2021; Svetlicic et al., 2023). These advancements have expanded the analysis toolkit for studying microbial pollutants and understanding microbial community dynamics under different environmental impacts (Zhang et al., 2021). In recent years, there has been an accelerated development of detection technologies such as biosensors (Petronella et al., 2022; Song et al., 2023). Highly sensitive molecular diagnostics, rapid response biosensor-based technologies and artificial intelligence integrated with machine learning (Yi et al., 2023) provide critical information for effective water quality monitoring, evaluation and action.

This review delves into the trajectory of waterborne pathogen bacteria detection methods over the past decade, considering their evolution in response to technological advancements and real-world applicability. This review also aims to offer a comprehensive and retrospective view of current trends in pathogen detection technologies in terms of their merits, constraints and identify their gap in on-site application. The review paper specifically focuses on waterborne bacteria that are identified as human pathogens and present significant risks to public health due to waterborne transmission. Pairwise comparison metrics and the compilation of the latest representative technologies and case studies also provide insight and a quick guide for readers to identify the advantages and limitations of various techniques for waterborne bacteria detection, with an emphasis on field-level applicability and timeliness analyses.

## 2 Current waterborne pathogen bacteria detection methods

### 2.1 Culture-based methods

Culture-based methods are regarded as highly standardized techniques as they have been employed for water safety monitoring (Tiwari et al., 2021). Central to the culture-based techniques is the propagation of bacteria in a nutrient-rich environment to establish their presence and quantities. Essentially, a water sample is placed on a growth medium, creating conditions conducive for the bacteria to multiply over time, then these bacteria form colonies that can be counted and identified, aided by staining and microscopy (Zulu et al., 2023). Microbiology has progressed substantially with the evolution of culture media, with Pasteur’s liquid medium in 1860 to Koch’s solid medium using agar (Bonnet et al., 2020).

One of the key aspects of culture-based methods is the use of selective media. Selective media are specially designed to promote the growth of specific bacteria while concurrently inhibiting others, as to ensure more accurate detection of target pathogens. For instance, the membrane filtration technique leverages this principle. By filtering water samples through a porous membrane, bacteria are retained and then placed on selective agar plates to facilitate the growth of desired pathogens and identify the specific pathogens. However, challenges arise when working with fastidious bacteria. These are microorganisms with very specific nutritional or environmental requirements, which makes it difficult to grow in standard culture mediums. They need more specific nutrient and atmospheric conditions, otherwise, they might be absent in the results, leading to underrepresented in research findings. This poses a significant limitation, especially when these pathogenic bacteria are of environmental importance.

Modern culture media replicate bacteria’s natural habitats by incorporating specific elements, enabling the cultivation of previously uncultivable bacteria. A relatively rapid culture method (72 h) was proposed by centrifuging samples and using modified charcoal–cefoperazone–deoxycholate agar (suppress the growth of background microbiota) to isolate *Campylobacter* more efficiently from poorly filterable water than the ISO 17995 standard (144 to 192 h) (Strakova et al., 2021). It also offers the potential for diverse bacterial detection from water samples.

Culture-based methods offer several advantages (Table 1). For instance, they can provide a clear picture of the viable bacteria in a sample since only living cells will reproduce to form colonies. Furthermore, they are cost-effective, and can be carried out even in basic laboratory settings, making them accessible to many researchers. This method (American Public Health Association [APHA], 2005) has been employed for monitoring of microbiological quality of water in cities with persistent waterborne disease outbreaks (Nienie et al., 2017). Membrane filtration and culture-based methods are recognized by the United States Environmental Protection Agency (US EPA) and widely regarded as gold methods for waterborne pathogen detection. Nevertheless, these methods also have some limitations. Culture-based techniques can also be time-consuming, as some bacteria may require days or weeks to form visible colonies. For example, the procedure required for incubating different bacteria was rather time-consuming, i.e., 4+24 h for *E. coli* bacteria analysis; 48+4 h for *Enterococcus* bacteria analysis; 72 h for aerobic mesophilic bacteria analysis (Nienie et al., 2017). This delay could be problematic when quick results are needed for public health decisions. Despite their limitations, culture-based methods remain crucial tools in waterborne pathogen detection. Ongoing advancements in bacterial culturing are anticipated to expand the understanding of the bacterial spectrum and provide deeper insights into various pathogenic detections.

**TABLE 1:** Comprehensive comparison of different detection methods in terms of their working mechanism, merits and limitations.

Method	Working mechanism	Merits	Limitations	References
Culture plating	• Dilution and spread of sample onto a solid growth medium • Colony morphology, counting	• Widely used and establish approach • Identify viable microbes • Cost-effective	• Time-intensive: requires longer incubation period • Limited to known culturable • Limited sensitivity for low-abundance microbes	Zulu et al., 2023
Membrane filtration	• Sample filtration through a membrane, followed by transfer onto a growth medium	• Concentrate microorganisms on the membrane for detection • Allow for enumeration and further analysis • Relatively shorter incubation periods compared to culture plating • Suitable for detecting low-abundance organisms	• Potential loss of organisms during filtration • Limited sensitivity for certain pathogens	Nienie et al., 2017; Ricchi et al., 2017; Brown et al., 2020
End-point PCR	• Amplification of a specific DNA sequence using primers, with the end result visualized after amplification	• Specific due to primer design • Reliable • Can amplify minute quantities of DNA • Established and widely used	• Only provide end result, no real-time monitoring • Contamination can result in false positives • Requires post-PCR processing for visualization • Limited quantitative capabilities	Deshmukh et al., 2016
Quantitative real-time PCR (qPCR)	• Amplification and quantification of DNA in real-time using fluorescent probes	• Rapid analysis with result available in real-time • High sensitivity, accuracy, and specificity • Wide dynamic range and broad application scope	• Limited ability to discriminate between closely related sequences. • High costs of instrument, analysis software and consumables • Susceptible to PCR inhibitors • Requirements for special skills and expertise on assay design and data analysis	Patel et al., 2016; Zhang and Ishii, 2018
Digital PCR (dPCR)	• Partitioning of the sample into numerous individual reactions, enabling absolute quantification	• Absolute quantification without the need for calibration curves • Improve precision and accuracy for low abundance target • Enhanced sensitivity for rare targets • Increase robustness against PCR inhibitors • Reduce reliance on standard curves or reference genes	• Limited dynamic range, typically lower than qPCR • Higher cost per sample due to the need for partitioning into numerous reactions • Higher susceptibility to false positives/negatives resulting from partitioning errors	Quan et al., 2018; Shi X. et al., 2021; Tiwari et al., 2022
Droplet digital PCR (ddPCR)	• Sample partitioning into numerous droplets, with amplification occurring in individual droplets	• Enhance precision and accuracy and more reproducible than qPCR • Superable for detection of target at low abundance • Reduce susceptibility to PCR inhibitors • Reduce reliance on standard curves or reference genes	• Limited dynamic range, lower than qPCR • Longer processing time compared to qPCR • High costs for instruments and reagents • Complex upon multiplexing	Mauvisseau et al., 2019
Loop-mediated isothermal amplification (LAMP)	• Nucleic acid amplification technique that uses multiple primers and an enzyme to rapidly amplify DNA at a constant temperature.	• Rapid assay (<1 h) • Isothermal (no need for a thermocycler) • Good selectivity and sensitivity • Simple assay setup and result readout • Low cost • Portable for field applications	• Challenge to perfectly design primers • Non-specific amplification and false-positive results caused by primer dimers and hairpins • Prone to cross-contamination upon final readout • Subjective due to visual inspection of reaction changes	Notomi et al., 2000; Martzy et al., 2017
Enzyme-linked immunosorbent assay (ELISA)	• Bind specific antibodies to the target bacteria, followed by the addition of an enzyme that produces a color change, which can be measured to determine the presence and concentration of the bacteria.	• Provide quantitative result • Versatility	• Cross-reactivity • Require pure samples • Time-consuming • Costly	Yadav et al., 2020; Campbell et al., 2021
DNA microarray	• Detection of target DNA/RNA using probe hybridization	• Simultaneous detection of multiple target genes • Increased assay capacity and scalability	• High background-level hybridization leading to reduced assay specificity • Lack of pathogen sequence information for probe design • Limited detection dynamic range • Not always quantitative	Gomes et al., 2015
Flow cytometry (FCM)	• Uses a laser to detect and measure physical and chemical characteristics of bacteria in a fluid stream	• Rapid detection • High sensitivity and specificity	• May not distinguish between viable and non-viable pathogens • Potential interference from background fluorescence may affect detection accuracy.	Safford and Bischel, 2019
Next-generation sequencing (NGS)	• High-throughput sequencing technologies that enable the parallel sequencing of millions of DNA fragments	• Ultra-high throughput • In-depth taxonomic characterization • Enhance entire microbial community profiling, and α- and β-diversity comparison • High accuracy	• Expensive sequencing equipment and high run cost • Less standardized and customized sample preparation procedures for different water types • Sequencing errors resulting from PCR bias • Intricate sequencing data processing • Requires proficient bioinformatics expertise	Tan et al., 2015; Kulski, 2016
Biosensor	• Biorecognition and signal transduction regarding change	• Rapid and real time detections with accuracy • Detection of broad spectrum of analytes in complex sample matrices • User friendly • High specificity, distinguish among various substances based on recognition materials • High selectivity regarding directed analyte • Offer versatile applications	• Temperature and pH influence sensitivity • Procedure of reagent preparation is extensive • Require regular monitoring and calibration for maintaining stability and accuracy over time • Limited life span	Srivastava et al., 2020; Naresh and Lee, 2021; Kadadou et al., 2022

### 2.2 PCR-based method

**FIGURE 1:**
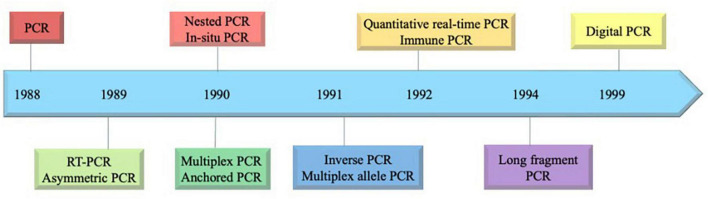
Development of PCR-based technology in chronological order.

The advent of the molecular technique, PCR, was first described by scientist Kary Mullis in 1985, revolutionizing molecular biology by enabling targeted DNA amplification (Saiki et al., 1985). In the context of water quality assessment, a significant milestone occurred in 2003, when a sequence-based molecular method was introduced to evaluate drinking water quality? (Hugenholtz and Tyson, 2008). Over the course, there have been a wide range of PCR-based methods, including quantitative PCR (qPCR), digital PCR (dPCR) and a combination of these techniques have been employed in waterborne pathogen detection. The development of PCR technology in chronological order is depicted in Figure 1.

Polymerase chain reaction analysis has been used to identify *E. coli* in primary water samples, stool samples and epidemics. The rapid advances in this field can be shown from a series of recent publications covering PCR analysis for waterborne pathogen screening in different sources, including drinking water and environmental samples (Miltenburg et al., 2020; Ambili and Sebastian, 2021) and the summary of PCR-based techniques of some of the representative research were depicted in Table 1.

#### 2.2.1 End-point polymerase chain reaction (PCR)

Conventional end-point PCR has been widely utilized since the 1990s for environmental surveys to determine the presence or absence of target pathogens in water. The PCR process typically involves three phases, which are denaturation, annealing and extension (Deshmukh et al., 2016).

Various waterborne pathogens have been detected using conventional PCR, including *E. coli O157:H7* (Saxena et al., 2015). *Salmonella* sp. and *V. cholerae* (Momba et al., 2006; Momtaz et al., 2013). The PCR technique is a valuable alternative to culture-based methods, addressing their time-intensive nature and limitations during emergency outbreaks. Notably, conventional PCR primarily provides qualitative results, indicating the presence or absence of the target.

On the other hand, a couple of challenges can lead to false-negative results in PCR assays designed for microbial detection. These include the limited sample volume that PCR reactions can accommodate and potential issues that arise during PCR processing. Traditional culture methods generally utilize a larger sample volume compared to PCR assays. Consequently, when pathogens are present in low quantities, PCR might fail to detect them, leading to false negatives. To address this limitation, DNA is typically extracted and purified before PCR amplification, which concentrates the total DNA from a more substantial sample volume (Yang et al., 2004). It is also worth noting that PCR inhibitors and other factors can also affect the sensitivity and accuracy of PCR.

Another drawback of PCR is its reliance on lab equipment settings, making on-site testing challenging. In terms of field application, the target samples collected on-site must be transported to central labs for analysis, which can be time-consuming, especially for samples from remote areas. In order to overcome logistical constraints and enable real-time genetic analysis in the field, portable and mini-PCR devices have been developed. A new portable PCR approach could allow the detection of specific genetic material in the samples while investigating pathogens in dynamic ecosystems or analyzing rapidly changing water conditions (Marx, 2015). These portable PCR devices range from prototypes to commercially available products. Some designers leverage standard PCR instruments for portability, while others have developed new miniaturized hardware. Some companies have taken the initiative to solve the mobility issue of PCR systems by developing handheld PCR instruments, as summarized by Li J. et al. (2020).

Some of the commercially available handheld PCR systems are Freedom 4 (Ubiquitome), Liberty 16 (Ubiquitome), miniPCR (Amplyus), Franklin (Biomeme), and Open quantitative PCR (Chai). They are small in footprint and light to carry on, where Biomeme’s handheld PCR Franklin is about the size of a soda can. Nguyen et al. (2018) demonstrated the potential of the Biomeme handheld qPCR system for rapid (<50 min) on-site detection and monitoring of *Flavobacterium psychrophilum* in filtered water samples. The study revealed that the results obtained with the Biomeme handheld qPCR system were comparable to those obtained with the conventional bench qPCR, indicating that field-based qPCR systems can effectively and promptly detect bacterial pathogens in water. The emergence of portable tools and methodologies has offered solutions to mobility challenges and empower researchers to identify unique genetic substances in their samples and facilitate quicker response to outbreaks and emergencies.

#### 2.2.2 Quantitative real-time PCR (qPCR)

Quantitative real-time PRCR (qPCR) revolutionized molecular diagnostics when it was introduced in the late 1990s. Since its commercialization in 1997, qPCR has emerged as a valuable technique for rapid and sensitive detection of waterborne pathogens (Saleem et al., 2023) and enable the detection and quantification of nucleic acids, such as DNA or RNA in real-time during the amplification process. The core principle of real-time qPCR involves the amplification of target DNA or RNA sequences, executed through specific primers and a DNA polymerase with exonuclease activity. This amplification process is tracked in real-time using fluorescent dyes or probes, which emit fluorescence upon binding to the amplified DNA. It enables the determination of the original target concentration by utilizing a standard curve created from known concentrations of gene constructs subject to serial dilutions (Zhang et al., 2021). Such a mechanism permits the direct detection and quantification of the target pathogen’s genetic material in water samples, circumventing the need for culturing and post-PCR analysis.

The US EPA introduced a standardized rapid method (known as US EPA method 1609.1), which uses qPCR for monitoring fecal indicators in recreational water ecosystems (USEPA, 2015). Such method enables quantification of *Enterococcus* in both marine and freshwater environments using qPCR technology. Saleem et al. (2023) reported employing a rapid qPCR-based method (US EPA method 1609.1) for monitoring *Enterococcus* bacteria at freshwater beaches in Canada and can be a reliable alternative to the traditional culture-based enumeration methods. The qPCR monitoring approach yields express same-day results since sample analysis takes only 4 h. The *Enterococcus* qPCR method can reduce the occurrence of inaccurate beach postings that are associated with the delayed results of the 24-h *E-coli* culture-based testing.

Notably, in the quantitative microbial source tracking of fecal pollution in water, host-specific genetic markers are continually being developed for the identification of fecal origin using qPCR (Vadde et al., 2019a). qPCR offers a quantitative analysis of the target samples and exhibits higher specificity as well as sensitivity compared to end-point PCR. It also allows high-throughput data processing without post-electrophoresis processes, making it less laborious. These advantages have made qPCR widely used in various research applications.

Despite its advantages in offering rapid and highly specific detection of target pathogens that are difficult to culture (Zhang et al., 2021), it is essential to note that quantifying pathogens using qPCR requires the creation of a calibration curve using known standards, such as plasmid DNA constructs, PCR amplicons, synthetic nucleic acids, genomic DNA, cDNA, or nucleic acids from biological samples (Botes et al., 2013). Ensuring the accuracy of calibration curves can be challenging, as errors in quantification may lead to biases in the measurement of molecular targets (Kralik and Ricchi, 2017). Additionally, protocol differences can impact the precision and consistency of qPCR measurements.

Apart from that, conventional qPCR has a limited ability to identify large targets. Recently, scientists have shifted their focus toward high-throughput qPCR techniques, such as nanoscale qPCR (ns-qPCR), to quantify multiple molecular markers within a single water sample (Brooks et al., 2020). This advancement allows for a more comprehensive analysis of the molecular composition in the sample. In terms of cost analysis, qPCR was the most cost-effective among molecular and culture methods, which only cost 52 Indian Rupees (0.62 USD) per sample. qPCR is considered as the best option for pathogens detection in drinking water with the highest sensitivity and lowest cost, compared to conventional culture method and end-point PCR.

Comparatively, qPCR has been observed to exhibit a superior detection sensitivity as highlighted by Ambili and Sebastian (2021), where a multiplex PCR and real-time PCR assay were developed for simultaneous seven waterborne pathogen detection. The assay detected DNA from all seven amplified genes without false positives or negatives, proving its exclusivity. In terms of detection sensitivity, qPCR had a detection limit (LOD) of 1 cell/mL, while PCR and multiplex PCR was 10–1000 cells. Such finding is consistent with Ramírez-Castillo et al. (2015), who reviewed the sensitivity of different PCR-based techniques. A SYBR green-based qPCR was used to monitor the presence of contaminants (*Enterobacter* sp.) in the environmental samples from potable water, surface water and river sediments in Lucknow City, India (Patel et al., 2016). The results demonstrated the high specificity of this method for *Enterobacter* detection in the collected samples.

Overall, qPCR enables fast and accurate quantitative analysis of specific organisms with exceptional sensitivity and specificity. qPCR is poised to remain a gold-standard molecular technique for various applications in the future. Advancements in technology will lead to significant reductions in equipment and consumable costs like fluorescent probes. At the same time, it is important to overcome the potential inconsistencies as well as biases. More developments are needed to enhance resistance to PCR inhibitors and further improve the portability of such devices to achieve more robust on-site testing.

### 2.3 DNA microarray

The microarray technology, which is better known as a lab-on-chip, or DNA chip, is versatile and can be employed for microbial detection. Lab-on-chips earn its name as it integrates multiple laboratory functions onto a small chip or substrate, enabling the miniaturization of laboratory processes. Microarray technology is a powerful tool for detecting multiple target genes in one assay. It involves a high-density arrangement of nucleic acid probes (genomic DNA, complementary-DNA, or oligonucleotides) organized in a two-dimensional matrix. Through the hybridization principle, the specific probe immobilized on the microarray interacts with the complementary target gene, leading to a fluorescent signal indicating the presence or absence of the target microorganism (Zhang et al., 2021). This simultaneous and efficient detection method has proven valuable in various research applications, where it offers a rapid and comprehensive view of the microbial community present in a sample.

DNA microarrays have been employed in various studies related to water microbiology. For instance, Inoue et al. (2015) utilized a DNA microarray as a screening tool to assess the presence of 941 bacterial pathogens in groundwater. In another study, a multigene target microarray was developed to evaluate the prevalence of waterborne pathogens in a fecal-contaminated watershed, revealing the presence of various viruses, bacteria, and eukaryotes (Weidhaas et al., 2018). Gomes et al. (2015) designed a DNA microarray with implanted probes targeting specific pathogens for water microbiological surveillance. Other studies focused on simultaneously detecting multiple pathogens in water samples, including bacteria, viruses and antibiotic-resistant strains (Bartley et al., 2019).

While DNA microarrays offer the advantage of simultaneous detection and high scalability, they also have some limitations. Non-specific cross-binding can result in high background noise, reducing specificity. Designing effective probes can be challenging, especially in the absence of complete pathogen sequence information. Other considerations include the high costs of experiments, limited detection dynamic range, and relatively lower sensitivity, accuracy and reliability compared to qPCR (Lemuth and Rupp, 2015; Sezer, 2021). Direct examination of waterborne pathogens using microarrays can be challenging due to their low concentrations. Some strategies to overcome this issue include prior culturing bacterial pathogens and increasing the volume of water samples (Gomes et al., 2015). These approaches aim to enhance the detection sensitivity and reliability of the analysis, ensuring a more comprehensive assessment of waterborne bacterial pathogens. Apart from that, according to Kostić and Sessitsch (2011), pre-PCR amplification is highly recommended to enrich target genes for improved detection.

Overall, microarray-based approaches for waterborne bacteria detection offer numerous benefits, including multiplexing, high sensitivity and automation. However, this technology also encounters some challenges related to probe design, detection of unknown pathogens and data analysis, therefore requires careful consideration of technical limitations. Modifications and additional steps may be necessary to enhance sensitivity and overcome the challenges of low pathogen concentrations in water samples.

### 2.4 Enzyme-linked immunosorbent assay (ELISA)

Enzyme-linked immunosorbent assay (ELISA) is an antibody-based technique for bacteria identification. It detects the surface marker on pathogens through antibody-antigen interactions (Campbell et al., 2021). ELISA is a widely acclaimed technique for pathogen detection. This approach uses antibodies to selectively attach to bacterial surface antigens.

The procedure involves coating microtiter plate wells with primary antibodies specific to the antigens of the pathogenic bacteria being analyzed. Subsequently, the sample suspected of containing the bacteria is introduced into the wells. When the pathogenic bacteria are present, the primary antibodies will bind to them, which capture the bacteria. Secondary antibodies conjugated with an enzyme are then added. These antibodies also specifically bind to the bacteria, forming an antibody-antigen-antibody sandwich. The addition of a substrate, i.e., the enzyme conjugated to the secondary antibodies catalyzes a reaction that results in a visible color change. The intensity of this color change is proportional to the concentration of the pathogenic bacteria and can be quantified using a spectrophotometer (Yadav et al., 2020).

There are several types of ELISA, including direct, indirect, sandwich and competitive (Crowther, 2009; Konstantinou, 2017). Each type of ELISA varies by the order of addition of components and the use of antibody or labeled antigen. For direct ELISA, sample antigens are attached to a solid surface. Subsequently, a conjugated antibody covalently linked to an enzyme is applied over the surface to bind with the antigen. Substrate enzyme will be added, and the reactions of enzyme will exhibit a change in color, which can be measured calorimetrically (Choudhury, 2020). It is straightforward, but less sensitive due to non-specific binding. Conversely, the indirect ELISA involves an additional procedure for amplification, where an unconjugated primary antibody binds to the antigen and then a labeled secondary against the host species of the primary antibody. The indirect ELISA is more sensitive owing to signal amplification but might induce cross-reactivity. Sandwich ELISA, which uses two antibodies for each antigen, is the most sensitive and is used to detect harmful bacteria in complex samples. Meanwhile, the more complicated competitive ELISA uses the standard anolyte plus an unknown amount of analyte to compete for antibody binding sites (Konstantinou, 2017). Competitive ELISA is utilized when the antigen is small or antibody binding sites are limited.

Overall, the ELISA method possesses numerous benefits, including requiring no pre-treatment, highly specific and sensitive ascribes to antigen-antibody reactions. ELISA is highly efficient as it allows for multiple simultaneous analyses without a complex sample preparation procedure. Besides, ELISA is safe and environmentally friendly since it does not use radioactive substances or large quantities of organic solvents. Nevertheless, this method also demonstrates certain drawbacks, for instance, it is laborious and costly to prepare antibodies because it is an advanced technique and requires relatively more costly culture cell media for the extraction of specific antibodies, long incubation time, requires highly trained personnel and advanced equipment, false positive or negative results, due to an inadequate surface blocking (Sakamoto et al., 2018; Yadav et al., 2020; Bhardwaj and Sharma, 2023). The benefits and drawbacks of ELISA for detecting pathogenic bacteria are summarized in Table 1.

Immunosensors have been miniaturized and automated in recent years to improve sensitivity, speed, ease of use as well as sample and reagent capacity. An innovative paper-based ELISA was developed for *E. coli* detection, which offers benefits over conventional methods, including quick detection (<5 h), lower cost (<1 USD/sample), ability to detect samples with 10^5^ cells/mL without advanced equipment and overall simpler execution (Shih et al., 2015). Similarly, Pang et al. (2018) developed a budget-friendly paper-based ELISA for detecting *E. coli* O157 H:7 and required only a small 5 μL sample and more rapid detection (<3 h), lower cost (∼0.066 USD/sample) with detection limit of 1 × 10^4^ CFU/mL. This approach has not been extensively employed in bacterial detection, but it shows promise for future applications given its improved portability and sustained sensitivity (Petrucci et al., 2021). Besides *E. coli*, several commercially available ELISA systems are designed for quick *Salmonella* detection. These include TECRA *Salmonella* from Tecra International Pty Ltd in French Forest, New South Wales, Australia; *Salmonella* ELISA Test SELECTA/OPTIMA produced by Bioline APS in Denmark and Vitek Immuno Diagnostic Assay System (VIDAS) by Biomerieux in Hazelwood, MO (Deshmukh et al., 2016). Technological advances are making assays more portable and affordable, improving their practicality.

### 2.5 Flow cytometry (FCM)

Flow cytometry (FCM) is an analytical method that has gained significant relevance in the identification and assessment of various types of water quality, particularly in the detection of pathogenic microorganisms. The fundamental principle involves the interaction between a laser light source and cells suspended in a liquid stream. The cells are aligned to pass single-file through the laser beam, resulting in the scattering of light and excitation of a fluorochrome, with signals sent to a computer for further processing (Safford and Bischel, 2019). FCM technique was first developed in the 1960s with limited application due to relatively high size thresholds for particle detection, non-specific binding of fluorescent stains, poor sensitivity as well as computational capacity (Wang et al., 2010). However, with the advent of more powerful and affordable instrumentation as well as the development of new analysis techniques, the applicability of FCM has expanded considerably. Nowadays, it is utilized in a wide array of fields, including water quality assessment and environmental monitoring.

Flow cytometry offers several advantages for pathogenic bacteria detection, including rapid, high-throughput analysis with high accuracy. FCM is capable of analyzing thousands of cells in a second, which allows speedy detection of bacteria in samples (Kennedy and Wilkinson, 2017; Cossarizza et al., 2019). Such technique also does not require DNA extraction and amplification. However, FCM possesses several limitations, such as non-portable, relatively high detection limit for certain bacteria, limited to liquid samples, complicated data acquisition and equipment, and requires skilled personnel (Wang et al., 2010). Moreover, the dependence on fluorescent dyes in FCM may lead to non-specific binding and photobleaching. The method may struggle at very low concentrations of bacteria detection without pre-enrichment. Furthermore, bacteria with similar physical and fluorescence characteristics can be difficult to distinguish, which limits the specificity. Besides, the expenses of equipment and reagents may hinder accessibility.

Some promising applications of FCM have been observed for pathogenic bacteria detection. The utilization of bacteriophage-based bioconjugates with bifunctional magnetic-fluorescent microparticles based on FCM technique was proposed for fast *E. coli* detection (Janczuk et al., 2017). The prepared bioconjugates obtained nearly 100% of the capture efficiency from 10 to ∼10^5^ CFU/mL, with a detection limit of ∼10^4^ CFU/mL due to FCM limitation. Similarly, FCM-based bacterial cell counts and fingerprinting techniques were employed by Van Nevel et al. (2017) for immediate follow-up of drinking water networks after maintenance, using *E. coli* counts as a benchmark to evaluate the FCM measurements and make decisions on returning the pipes to service. The application of FCM for immediate follow-up of drinking water networks after maintenance could reduce waiting times and drinking water losses.

While conventional FCM cell sorters operate through the identification and quantification of certain biomarkers on or within a cell, Schraivogel et al. (2022) innovatively combined the spatial resolution of fluorescence microscopy with flow cytometric cell sorting, establishing a high-speed image-enabled cell sorting that records multicolor fluorescence and can capture multiple images of individual cells flowing through the system at a remarkable speed of 15,000 cells per second. It was previously unfeasible to sort individual cell at this speed, but this breakthrough allows rapid identification and isolation of cells, as well as extensive ramifications and experimental strategies. With rapid, precise and reliable analysis, FCM holds immense potential to ensure safe and healthy water systems.

## 3 Emerging pathogen detection methods

### 3.1 Digital PCR (dPCR)

Environmental microbiologists have recently embraced digital polymerase chain reaction (dPCR) as a third-generation PCR tool to quantify microorganisms in aquatic and terrestrial ecosystems (Borchardt et al., 2021). The utilization of dPCR has experienced rapid growth, primarily driven by its increasing application in environmental monitoring and characterized by its unique mechanism that involves dividing a sample into numerous individual reactions (Lei et al., 2021). This partitioning step creates thousands of mini-reactions within the sample. Following thermal cycling, the presence or absence of the target gene is determined in each partition. Finally, the absolute copy number of the target gene can be calculated by analyzing the positive and negative partitions via the principles of Poisson distribution law (Shi X. et al., 2021).

Numerous studies were reported on the use of dPCR for different water bodies surveillance, including wastewater, surface water and drinking water. The most frequently used platform was Bio-Rad QX200™ (Bio-Rad Laboratories), QuantStudio™ 3D (Thermo Fisher Scientific), Bio-Rad QX100™ (Bio-Rad Laboratories), QIAcuity (QIAGEN) and BioMark™ HD (Fluidigm) systems, which rank in decreasing order (Tiwari et al., 2022). The presence of PCR inhibitory substances in sediment samples poses a challenge when using qPCR (Singh et al., 2017). Even small amounts of these inhibitors can significantly delay the threshold cycles in complex samples, leading to inaccurate estimates of the template copy number (Sidstedt et al., 2020). The dPCR can perform absolute quantification of target nucleic acid, which has limited ability to overcome the shortcomings of qPCR.

Most of the studies demonstrated comparable dPCR measurement of molecular targets compared to qPCR measurement, with dPCR systems usually exhibiting enhanced sensitivity, precision, and lower inhibition rates (Lei et al., 2021). Notably, careful optimization of primer or probe concentrations and thermocycling conditions is necessary for genetic targets assay. Comparable results were observed across various types of microorganisms, with many studies recommending the use of dPCR over qPCR for microbial quantification in wastewater. The lower limit of quantification (LOQ) obtained through dPCR in these studies suggests its potential usefulness in wastewater surveillance, particularly for detecting pathogens present at trace levels. The utilization of dPCR platforms may lead to increased costs and longer processing times compared to qPCR. However, the substantial improvements in analytical performance, particularly in sensitivity and accuracy, make dPCR a promising alternative for quantifying pathogenic microorganisms in aquatic environments (Tiwari et al., 2022). Wastewater analysis using dPCR holds significant promise as a robust and sensitive method for microbial contaminants monitoring.

Nevertheless, Shi X. et al. (2021) reported higher levels of inhibition in dPCR measurements compared to qPCR when analyzing wastewater samples, which was inconsistent with most studies. Apart from that, dPCR has a relatively longer preparation and processing time compared to qPCR. These varying observations might be attributed to the specific platform used or the characteristics of the water matrix being analyzed. It is essential for future research to conduct comprehensive comparisons of performance characteristics among different dPCR platforms and to investigate further the impact of inhibition on dPCR measurements in wastewater samples.

In terms of cost, dPCR instrument is relatively more costly, which result in higher usage cost. The use of dPCR for routine microbial water quality monitoring may be limited by cost and may not be practical at this stage. However, it remains valuable for research purposes and ensuring qPCR control materials’ accuracy. Regarding efforts to make dPCR more accessible, future research should focus on better understanding key measurement factors, comparing different dPCR platforms, and finding ways to reduce costs. These efforts will help make dPCR more affordable and practical for broader applications in water quality monitoring.

#### 3.1.1 Droplet digital PCR (ddPCR)

Droplet digital PCR (ddPCR) and chip digital PCR (cdPCR) are sub-categories of digital PCR with ddPCR offering higher precision and high-throughput capabilities. ddPCR has emerged as a powerful tool for absolute DNA quantification, eliminating the need for standard genetic references and being widely utilized in pathogen detection (Lei et al., 2021). The third generation of PCR technology, ddPCR, partitions a DNA template into numerous water-oil emulsion droplets before PCR amplification, with the final signal readout occurring at the endpoint of amplification. Since its launch in 2011, ddPCR has garnered significant attention and has been broadly employed in various disciplines, including clinical as well as environmental research (Mauvisseau et al., 2019).

Due to its increased sensitivity, accuracy and reproducibility, water emulsion-based ddPCR has emerged as a potential alternative technology to overcome qPCR’s inhibitory effects (Hindson et al., 2011; Taylor et al., 2017). Falzone et al. (2020) used it to rapidly detect waterborne bacteria *L. pneumophila* in a vitro model of water heat treatments. Despite high sensitivity, ddPCR was able to accurately detect low concentrations of L. pneumophila, demonstrating its superior accuracy and sensitivity compared to RT-qPCR. Such highly precise techniques are essential, especially in high-risk areas like healthcare centers, to eliminate lengthy waiting periods associated with culture-based methods, offer quick detection and avoid any possible outbreak of Legionnaires’ disease.

Singh et al. (2017) detailed the first comparison between ddPCR and qPCR on *Salmonella* and its application for river sediments. One notable advantage of ddPCR is its capability to achieve absolute DNA quantification without relying on external calibrators (Pinheiro et al., 2012). In ddPCR, the sample is partitioned into multiple individual reaction chambers containing one or no copies of the target sequence before undergoing PCR cycles (McDermott et al., 2013), allowing more precise and reliable quantification results.

The partitioning of the sample in ddPCR, forming multiple droplets, significantly reduces susceptibility to PCR inhibitors (Morisset et al., 2013). Researchers have developed a duplex ddPCR platform that detects *E. coli O157* and *Listeria monocytogenes* simultaneously and sensitively (Bian et al., 2015). Mineral oil-saturated polydimethylsiloxane chip were used in this platform to prevent droplet evaporation and differentially labeled fluorescent probes. Its great sensitivity outperforms traditional qPCR as it was able to detect both bacterial strains with a low detection limit of 10 CFU/mL within 2 h. It was worth noting that it is only applicable in a laboratory setting, and further improvements are needed.

Apart from that, Falzone et al. (2020) established the superiority of ddPCR over RT-qPCR in detecting *Legionella pneumophila*, a harmful bacterium prevalent in water systems, particularly in hospitals. The ddPCR method demonstrated greater sensitivity and accuracy, especially with low bacterial loads and in fragmented DNA. It also showed higher accuracy in tracking the effectiveness of heat shock treatments. The researchers recommend ddPCR for rapid detection and monitoring of *L. pneumophila* in high-risk environments like healthcare centers. They also suggest its potential use in clinical settings for early detection and treatment assessment, which could improve health and socio-economics.

The study of Cao et al. (2015) revealed a high correlation between ddPCR and simplex qPCR (coefficients >0.93, *p* < 0.001) on fecal water assessment. The study also revealed three main limitations of ddPCR, including the need to dilute samples, especially in highly polluted water samples, to achieve exact quantification. Second, analytical sensitivity variability, where ddPCR sensitivity depends on the number of droplets per reaction, resulting in different detection limits from reaction to reaction. When analyzing non-detect data, especially for markers like human-fecal HF183, this variability should be acknowledged. The third limitation was about partitioning issues, and the study found that some scenarios may affect the partitioning of molecular targets into the water-in-oil droplets during ddPCR. Linkage of targets can underestimate copy counts by linking several copies of particular genes in one droplet. High total DNA concentrations may require dilution or pretreatment to resolve partitioning issues. Even with inhibitor doses one to two orders of magnitude higher than qPCR, ddPCR is quantified accurately.

Digital PCR has seen greater adoption in clinical settings compared to environmental ones, primarily due to cost constraints and the complexity of environmental samples. Environmental applications often involve large-scale testing of various samples, making the cost per reaction for dPCR relatively higher. Moreover, environmental samples can be complex and may contain inhibitory substances, requiring additional optimization procedures that increase cost (Tiwari et al., 2022). Overall, the advantages offered by ddPCR improved sensitivity, precision and reproducibility, along with its ability to overcome PCR inhibitors and provide absolute quantification, make it a valuable tool for molecular diagnostics. As operating system and chemical reagent costs decrease and assay throughput and dynamic range grow, ddPCR could become a routine test method like qPCR in environmental studies.

### 3.2 Loop-mediated isothermal amplification (LAMP)

The loop-mediated isothermal amplification (LAMP) method was introduced by Notomi et al. (2000), and achieves DNA amplification at a constant temperature without a thermal cycler. It employs 4–6 primers that recognize 6–8 target DNA regions, along with a DNA polymerase that synthesizes DNA while displacing the strand. This unique process forms a loop structure that enables exponential amplification under isothermal conditions (60–65°C). The reaction’s progress can be observed through turbidity, fluorescence changes (Notomi et al., 2000). LAMP has several advantages, e.g., simple operation and low-effectiveness (no need for bacterial cultivation or complex extraction) and high specificity compared to conventional detection methods. LAMP assay was 10 to 100-fold more sensitive than PCR with a detection limit of 10 or less template (Notomi et al., 2000). This technique incubates the mixture of primer, gene sample, DNA polymerase and substrates with strand displacement activity at a constant temperature for one-step amplification and detection (Li et al., 2017). The results can be visually interpreted using a low-cost turbidimeter, that ensures reliable target gene sequence detection.

While LAMP technology offers advantages in nucleic detection, it also has drawbacks that limit its application. The primary challenge lies in the primer design as it needs 4–6 different primers. These must be carefully designed for high specificity, which makes the process of primer design challenging and time-consuming. Despite high specificity, LAMP is not completely immune to the risk of non-specific amplification. Using multiple primers inherently risks amplifying non-target sequences, which can lead to false-positive results (Martzy et al., 2017). This can have serious implications in environmental testing scenarios where accurate detection is critical. Another limitation of LAMP is the risk of product carry-over causing contamination. Given the high amplification efficiency of LAMP, even minor contamination can lead to false-positive results. Despite these drawbacks, ongoing research and development efforts continue to refine the method and mitigate limitations.

Research on LAMP has made significant progress in enhancing its applications for waterborne pathogenic detection. LAMP has been effectively integrated with various detection techniques, e.g., microfluidic platforms and most probable number (MPN) approaches, enabling rapid, sensitive and field-deployable detection systems for identifying waterborne pathogens. Ahmad F. et al. (2017) and Ahmad R. et al. (2017) integrated most probable number (MPN) with LAMP for the direct detection of *E. coli* and *Enterococcus feacalis*. It eliminates the need for nucleic acid extraction and can amplify directly from bacterial cells at 63°C. The method was adapted to a microfluidic platform, achieving up to 800 signal-to-noise ratios, sensitivity under 10 CFU, and detection time of ∼20 min. This MPN-LAMP method shows potential as a rapid, efficient and field-ready system for detecting waterborne pathogens. In another study, Fu et al. (2021) developed a quick on-chip gene quantification method using LAMP PCR to measure fecal indicator bacteria (FIB) genes, e.g., *E. coli* and *Enterococcus* spp, through an MPN approach.

This colorimetric LAMP assay permits the detection of as few as 4 gene copies per well and correlates strongly with qPCR analysis. This assay was further applied to quantify FIB in different water environments, including freshwater reservoirs, beaches, farms and sewage, showing that MPN-LAMP method can quickly and easily quantify environmental FIB genes without expensive qPCR instruments. Besides, Jin et al. (2021) combined microfluidic chip with LAMP technology for rapid detection of multiple waterborne pathogenic bacteria (within 35 min) with a detection limit between 7.92 × 10^–3^ and 9.54 × 10^–1^ pg of bacterial DNA per reaction with sensitivity and specificity of 93.1 and 98.0%, respectively. The advancements in real-time LAMP systems have enabled quantitative assessment of pathogen concentrations in water samples, bringing laboratory testing closer to the field.

### 3.3 Next-generation sequencing (NGS)

Next-generation sequencing (NGS) is an emerging technology in the field of genomics that enhances bacteria identification in environmental and biological samples (Garrido-Cardenas et al., 2017). Unlike conventional sequencing methods analyze one gene at a time, the NGS technique encompasses whole-genome sequencing, metagenomics and amplicon sequencing and revolutionizes the field with simultaneous sequencing of millions of DNA.

Whole genome sequencing offers high-resolution information on individual bacterial strains (Quick et al., 2015), while metagenomics overcomes the limitations of culture-dependence by the ability to capture the complete genetic content of the microbiome for identification, including unculturable bacteria (Li et al., 2015). As for amplicon sequencing, it predominantly targets the 16S rRNA gene and offers a relatively cost-effective approach for taxonomic profiling of microbial communities (Quince et al., 2017). The advantages of NGS technique are quick sample preparation (4–6 h), no PCR step in the preparation step, which can reduce bias and error, fast turnover rate and runs can be finished within a day, where the average read length is longer than that of any second-generation sequencing technology (Tanchou, 2014). Nevertheless, there are some drawbacks, e.g., high operational costs, the need for skilled expertise, complex data analysis and lack of standardized protocols. With the rapid evolution of technology, the merits of NGS are increasingly evident, despite the inherent challenges (Garner et al., 2021). Some useful websites related to NGS tools, browsers, portals, providers, and online database has been listed and reviewed (Kulski, 2016).

The NGS platforms like single-molecule real-time sequencing technology (SMRT) can produce sequences exceeding 1000 bp read lengths and have surpassed those generated using Illumina and Roche 454 (Tan et al., 2015). A novel single molecule SMRT based on nanopore technology, MinION has garnered significant interest due to its cost-effectiveness and portability. The device could achieve single read lengths of up to 5500 bp in a single run. However, it also exhibited a relatively high sequence error rate of approximately 30% (Tan et al., 2015). Nanopore sequencing shows great potential for online waterborne nucleic acids detection ascribed to its high portability and ability to provide real-time data and foster the development of real-time sensing platforms for water-quality monitoring.

In recent years, NGS has been extensively applied to detect waterborne pathogenic bacteria (Supplementary Table 1). Vierheilig et al. (2015) evaluated the potential of NGS for water quality assessment, specifically for detecting fecal pollution in backwater catchment areas in Vienna and deduced that NGS techniques hold substantial potential for various aspects of water quality surveillance. However, the study suggested that the amplicon sequencing of broad bacterial communities might lack the requisite sensitivity for identifying pathogens or fecal indicators present in small quantities in the environment given the currently applied sequencing depth. Meanwhile, Vadde et al. (2019b) utilized NGS on the Illumina platform to analyze the bacterial community structure of the Tiaoxi River water, targeting bacteria that are either fecal-associated or pathogenic. Using water, fecal and wastewater samples, it determined the presence of potential pathogens and fecal bacteria, revealing spatial and temporal variations in bacterial diversity. The presence of pathogenic genera, e.g., *Acinetobacter*, *Aeromonas*, *Arcobacter*, *Brevundimonas*, *Enterococcus*, *Escherichia-Shigella*, and *Streptococcus* in various locations implies potential health risk. The findings suggested that 16s rRNA genes targeted NGS is a crucial tool for initial environmental sample screening.

16S NGS technique was employed to detect enteric pathogens and evaluate bacterial compositions in wastewater treatment plants (Greay et al., 2019). The V4 region of the 16S rRNA gene was sequenced on the Illumina MiSeq platform. A limitation was observed in misidentifying 16S sequences from chloroplasts as Cyanobacteria within the Greengenes database. To address this issue, the authors suggested caution and validation through comparison with the NCBI nr/nt database. In another study, Svetlicic et al. (2023) employed whole-genome sequencing and identified four potential new species in the *Legionella* genus by characterizing 39 isolates collected from different countries during environmental surveillance in building water systems. Functional annotations were also performed on the genomes, including virulence and antimicrobial resistance, and provided insights into their pathogenic potential and genetic relatedness.

Meanwhile, NGS was paired with a pathogen database and thoroughly analyzed 41 water samples from urban rivers and sewage plants in Changzhou City, China (Cui et al., 2019). It expanded the pathogen species database across 23 genera to reduce false positives and aid in precise identification, particularly enteric (e.g., *Arcobacter*, *Bacteroides*) and environmental (e.g., *Acinetobacter*, *Aeromonas*, and *Pseudomonas)* species. Furthermore, the study also employed qPCR to quantify specific pathogens/indicators, revealing the dominant species like *E. coli* and *Enterococcus faecalis*. More recently, a study by Nakanishi et al. (2023) used whole genome sequencing to investigate an outbreak of *Legionella pneumophila* at a bath facility in Japan. It also underscores the significance of comprehensive genetic analysis of environmental and clinical isolates during such outbreaks. With a concerted effort to overcome existing barriers, the understanding of microbial roles and activities facilitated by NGS could revolutionize industry practices and benefit both water quality and public health. As researchers continue to address challenges and explore innovations, the future perspectives for pathogenic bacteria detection with NGS remain promising.

## 4 Biosensors

**FIGURE 2:**
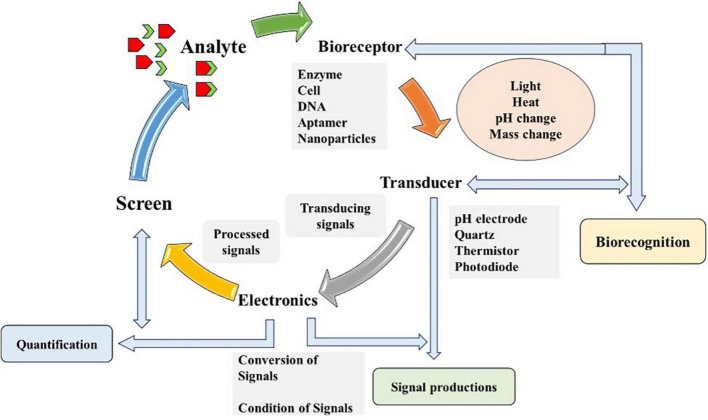
Schematic diagram illustrating the working principle of biosensor. The diagram outlines the detection process of biosensor consists of four components and steps: (1) Bioreceptor (based on enzyme, cell or DNA), which selectively binds the target analyte (chemicals, microbes, and organic or inorganic agent); (2) Transducer, which converts the bioreceptor-target analyte interaction (light, heat, or change) into measurable signals; (3) Electronics or the signal processor, which amplifies and transform signals into a readable output; and (4) Output display or screen, which provide real-time, qualitative or quantitative information about the target analyte presence and concentration in the sample. Diagram modified from Das et al. (2022).

Biosensors are analytical devices that convert biological responses and amplify them into electrical signals (Cammann, 1977) coined the term biosensor, and the International Union of Pure and Applied Chemistry (IUPAC) introduced its definition. Biosensors should be extremely definite and sovereign of physical constraints, for instance, temperature, and pH and must be reusable. Multidisciplinary research is required for immobilizing procedures, transducing strategies and fabrications (Thévenot et al., 1999). Biosensors are based on biorecognition layers and transducers, playing a significant role in measuring and quantifying biomarkers. Various recognition materials include ligands, antibodies, enzymes, nucleic acids, tissues, microorganisms, organelles, cell and biomimetic receptors. A sensitive transducer (electrochemical, optical, piezoelectric, and magnetic) converts biochemical signals (analyte identification and quantification) that takes place on recognition layer (Kaya et al., 2021). Real-time analysis of sensitive and selective targeted analyte in a rapid and cost-effective manner is possible with biosensors. Detection via biosensors follows three phases, analyte identification, signal generation as a result of biochemical reaction and its quantification (Nikhil et al., 2016) as shown in Figure 2.

Back in 1956, Leland Clark developed an oxygen probe known as Clark electrode and later, in 1962 he described “how to develop electrochemical sensors further intelligent.” Since that time, the interest of researchers explored various techniques, transducers and bioreceptors to achieve maximum specificity, selectivity, sensitivity and stability of biosensors. Innovation and development of biosensors from 1972 to 1992 are summarized in Supplementary Table 2. The last two decades can be witnessed as a period of rapid development and diversification of biosensor concepts, principles and applications. Biosensors are still a broad and active area of research and development, bridging principles and concepts of basic sciences with fundamentals of electronics, nano and micro technologies.

### 4.1 Generation and characteristics of biosensors

Based on response there are three generations of biosensors, (i) response based on diffusion, (ii) response based on adding mediating agents, and (iii) response directly produced by the sensor (Karunakaran and Bhat, 2015).

Effective and efficient biosensors are characterized by selectivity (response to specific target analyte), sensitivity (unresponsive to other interfering agents), range (analyte concentration for sensor to be operative), response time (time to sense actual, target and analyte concentration), stability (change in baseline over the fixed period), reproducibility (accuracy: sensor output to be achieved), detection limit (minimum concentration of analyte on which sensor generate biochemical signals), and lifetime (effective and responsive to target analyte each time) (Tang et al., 2004).

### 4.2 Types of biosensors

Variations in accuracy, detection limit, sensitivity and robustness can be observed in each biosensor. These variations are based on the working mechanism of biosensors. For instance, broad spectrum detections can be made possible with optical biosensors [Fiber optics, surface plasmon resonance (SPR), and resonant mirror] along with accuracy, ease of operations and sensitivity (Velasco-Garcia, 2009). Merits of biosensors include stability and reliability as well as not affected by noise, heat and electromagnetic waves. These biosensors are fabricated with low-cost materials, have high specificity and can be integrated on a single chip. While the limitations are brittle fiber tip, photobleaching and complex calibration (Chen and Wang, 2020; Uniyal et al., 2023). Magainin I with silver nanoparticles enhances SPR sensitivity for detection of *E. coli* (Singh et al., 2015). Similarly, chitosan-modified quantum dots as fluorescence with label fluorescence sandwich assay biosensor were used for the detection of *E. coli* (Dogan et al., 2016). Evanescent wave fiber biosensor with DNA as biorecognition materials were used by Xiao et al. (2014) for Shigella and *E. coli* detections. However, biorecognition materials, including aptamers with fluorescence-based biosensors (Zhang et al., 2016), antibodies with chemiluminescence-based biosensors (Wolter et al., 2008) and cysteine as cross-linker along with gold nanoparticles (Safavieh et al., 2013) with calorimetric biosensors were used for detection of *E. coli*, *Salmonella*, and *Staphylococcus*.

Piezoelectric/quartz crystal microbalance (QCM) biosensor detect changes in resonance frequency and mass on the crystal surface of biorecognition material as a result of antigen-antibody binding events. Frequency-dependent piezoelectric materials are utilized for the generation of acoustic waves (Babacan et al., 2000; Bridle and Desmulliez, 2021). These biosensors are user-friendly, economical, label free, and provide online analysis for antibody-antigen interactions. Drying and washing steps, higher incubation times and regeneration of crystal surface are some demerits (Rao et al., 2006). DNA-based detection and gold nanoparticles with QCM as amplifications materials (Wang et al., 2008), antibody-functionalized gold nanoparticles with QCM (Guo et al., 2012), immunoaffinity layer bound of antibodies with acousto-gravimetric flexural plate wave transducer (Pyun et al., 1998) were used for detection of *E. coli*.

Electrochemical biosensors have proven their tenacity and play a significant role in detecting pathogens in the available biosensors. The working principle of these biosensors is based on changes in capacitance, resistance and conductance. Oxidation-reduction takes place on the surface of biorecognition layer because of analyte’s binding activity, offering signals for sensing. These biosensors mostly rely on enzyme-catalyzed reactions to induce potential difference (Nayak et al., 2009; Bridle and Desmulliez, 2021). Polymeric nanocomposites and screen-printed interdigitated microelectrodes with electrochemical biosensors were used for detection of *E. coli* (Xu et al., 2016). The associated advantages of electrochemical biosensors include low cost of testing, portable, automation, low limit of detection, higher sensitivity, unaffected by sample turbidity and interference from fluorescence and absorbance, simple instrumentation and require low power. Exhibit superior sensitivity, and sophisticated linear detection in an extensive array of samples, temperature and electrolytes over other biosensors. The ionic strength of biofluids and pH can greatly influence the response of these sensors, reproducibility and stability, limited shelf life and cross-sensitivity of other gases are some associated drawbacks (Thévenot et al., 2001; Grieshaber et al., 2008; Srivastava et al., 2020).

Voltametric biosensors measured change in current and potential for detection of analyte. Differential pulse voltammetry, cyclic voltammetry and square wave voltammetry techniques are commonly used for environmental analytes (Reddy et al., 2014). Avidin-modified polyaniline (PANI) deposited electrochemically onto a platinum (Pt) disc electrode (Arora et al., 2007) and 1-fluoro-2-nitro-4-azidobenzene modified octadecane thiol (Pandey et al., 2011) with voltametric biosensors for detection of *E. coli*. Potentiometric biosensors consist of reference and working electrodes. Usually, enzyme is coated on working electrode as bioactive materials. Ionic species are exchanged during enzyme-catalyzed reactions. These biosensors are based on ion selective electrodes and output signal is generated as a result of ions accumulation (Srivastava et al., 2020). The natural affinity between streptavidin and biotin was used for the detection of Bacillus subtilis along with potentiometric biosensor (Uithoven et al., 2000). Generation of current can achieve detection of microorganisms in response to electrooxidation/reduction by enzyme on biorecognition materials surface of working electrode in amperometric biosensors. Conducting polymers, graphite, noble metals and modified carbon are commonly used as working electrodes (Singh et al., 2014). Single-stranded DNA (ssDNA) is utilized in the detection of *E. coli* RNA by microelectromechanical system (MEMS) based biosensors (Gau et al., 2001). Gravimetric biosensor measures mass variation at diverse frequencies, and sense pathogens and antigens by binding interactions (Atalay et al., 2018; Cali et al., 2020). Meanwhile, thermal biosensor detects heat change owing to biological reaction (Lakshmipriya and Gopinath, 2019; Vasuki et al., 2019).

**FIGURE 3:**
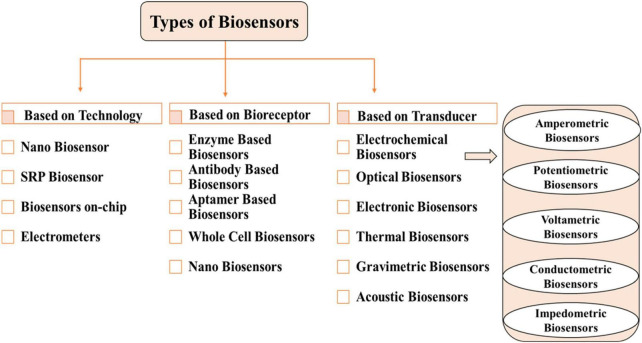
Types of Biosensors based on technology, bioreceptors and transducers.

Biosensor classification is a multifaceted and interdisciplinary field. Types of biosensors are classified based on biorecognition layer, transducers, technology and detection system are shown in Figure 3.

Enzymes, antibodies, aptamers and whole cell based biorecognition/bioreceptor layer uncover target analyte based on several mechanisms of binding and catalytic reactions. The performance of these bioreceptors can be enhanced for sensing cancer biomarkers, pathogens, toxins, proteins, cells and spores by using various transducers and nanomaterials (Kaur and Shorie, 2019; Singh et al., 2020; Bhardwaj et al., 2021; Öndeş et al., 2021).

Nano biosensors use nanoscale materials such as nanoparticles (NPs), nanowires (NWs), nano rods (NRs), carbon nanotubes (CNTs), quantum dots (QDs) and nanocomposites for quantification of biological or chemical signals, also enriching performance and detection power (Abdel-Karim et al., 2020; Shi S. et al., 2021).

### 4.3 Biosensors for detection of waterborne pathogens

Infections from pathogens are responsible for various diseases and human loss worldwide. Prevention is possible through timely and accurate detection of causative pathogens (Mao et al., 2020). It is a pressing priority to detect and identify pathogens in water and food products, ensure the health and quality of consumable resources, and reduce economic losses. World Health Organization (WHO) documented that most diseases, including dysentery, cholera, infections from worms, diarrhea and skin infections (Water Quality and Health Strategy 2013–2020), are due to waterborne pathogens. Annually, almost 7.15 million people get infections, 6,630 fatalities and more than USD 3.3 billion in economic losses to the healthcare system due to waterborne pathogens (World Health Organization [WHO], 2013; Collier et al., 2021). Along with innovation and advances in the medical field, the recent COVID-19 pandemic highlighted that infectious diseases are still a major challenge for healthcare systems around the planet. Globally, 15% of fatalities are caused by contagious diseases by World Health Organization [WHO] (2016).

Therefore, it is crucial for human health to develop a fast, accurate and selective system that can detect (quantitative and qualitative) pathogens in water. Various treatments and advancements in the detection system of pathogens have been documented in the past decade (Dietvorst et al., 2020; Lin et al., 2020). These methods are highly sensitive, labor-intensive, time-consuming, relatively expensive and limited to laboratory environments (Kaya et al., 2021).

The detection and quantification of specialized biomarkers of infectious diseases can be achieved with high sensitivity and selectivity in rapid and economical manners with biosensors. Biosensors provide real-time and *in situ* analysis of targeted analyte, without costly and complicated sample preparation. Additionally, it can be utilized in forensics, point-of-care diagnostics, medical, biomedical research, discovery of drugs, food quality, and environmental samples (Nikhil et al., 2016).

Nowadays, biosensors which are highly selective, sensitive and specific, employed for rapid detection of pathogens in water without any enrichment or spiking. These sensors are fast, small, portable, and can achieve maximum probabilities. It can also be used with digital tools like smartphones and is better for low-income states (Li et al., 2019; Poghossian et al., 2019). The specificity of biosensors is related to bioreceptor materials. Biochemical identification mechanisms are based on bioreceptor (bio-molecule) elements. These receptors are involved in binding of analyte to the sensing segment of biosensors. Supplementary Table 3 summarizes a list of broadly used bioreceptor for the identification of pathogens.

The presence of pathogens in various environment segments, especially in hydrosphere, such as protozoans, fungi, viruses, viroid and bacteria, are the major sources of spreading infectious diseases and pandemics. It is possible to combat the spreading of contagious diseases caused by these pathogens via biosensors, based on transducers and nanotechnologies for fast, sensitive, on-site and specific detections. Declining quality, quantity and increasing demand of the population for water, various pathogen detection modes, including optical, fluorescence, SPR, electrochemical, luminescence, nanotechnologies, colorimetry, or a combination of detection modes, can be utilized for public safety (Xu et al., 2018; Dey et al., 2020). Table 2 summarizes various kinds of biosensors used for detecting pathogens in water.

**TABLE 2:** Biosensors used for detection of pathogens in water.

Detection method/ sensor	Target recognition molecule	Substrate material	Signal transducer	Sample matrices	Pathogen type	Detection time	Absolute or relative quantification	Detection limit	References
Faster R-CNN (region-based convolutional neural networks)	DNA signals	(Artificial intelligence) AI-biosensing: microscopic imaging of bacteria	Fluorescence	Real world water samples	Escherichia coli exposed to T7 phages	5.5 h	Absolute	10 CFU/ml	Yi et al., 2023
CHANCE (Cas12a-HCR evANescent wave fluorescenCE)	crRNA *with rfbE* gene of *E. coli* O157:H7	Fiber evanescent wave fluorescence	Optical: fluorescence	Water	*E. coli* O157:H7	50 min	Absolute	17.4 CFU/ml	Song et al., 2023
Interdigitated transducer (IDT) electrodes covered with Ti/Au layer	Antibody	Enzyme linked immunosorbent assay (ELISA)	Piezoelectric, Mass sensitive: Surface acoustic wave	Water	*Legionella pneumophila*	1 h	Relative	2.01 × 10^6^ CFU/ml	Gagliardi et al., 2023
Fabrication: stacking and drawing; photonic crystal fiber coated with stable thin layer gold	Pathogens: bacteria	Fiber: photonic crystal fibers	Optical: surface Plasmon resonance (SPR)	Pure water	Vibrio Cholera, *Escherichia coli*, Bacillus Anthracis, and Enterococcus Faecalis	Not specified	Relative	3714, 4827, 4502, and 5161 nm/RIU	Haque et al., 2022
Protein/G/thiol monolayer, microfabricated gold WE and the anti-*Cryptosporidium* antibodies	Antibody	Fabrication: Thiolated protein/G and specific anti-*Cryptosporidium* monoclonal antibodies (IgG3).	Electrochemical: impedance	Water	*Cryptosporidium* Oocysts	24 h	Absolute	∼ 20 oocysts/5 μL	Luka et al., 2022
Fabrication of Au NR-based substrates: Immobilization and functionalization	Bacterial cell: specific antigen-antibody reaction	Nano materials: citrate-capped gold nanorods	Optical: localized surface Plasmon resonance (LSPR)	Water	*Escherichia coli*	30 min	Absolute	8.4 CFU/ml	Petronella et al., 2022
Bio-TFTs (Thin film transistors)	Antigen-antibody reaction	Fabrication: ZnO Thin film transistors on (PET)	Electrochemical: voltametric	Deionized water	*Escherichia coli*	3–10 s	Absolute	2 × 10^–10^ A at 5Vgs	Salinas et al., 2022
Microfluidic microchannels with positive- (p-DEP) forces	Antibody	Inter digited electrodes coated with activated gold	Electrochemical: Impedance	Tap water and waste water	*Salmonella*, *Legionella*, and *E. coli 0157:H7*	30–40 min	Absolute	3 bacterial cells/ml	Muhsin et al., 2022
Fluorescence-based ATmega328P prototype sensing	Aptamer I Conjugated with hydrophilic SPIONs and Aptamer II conjugated Cadmium telluride-mercaptopropionic acid quantum dots (CdTe-MPA QDs)	Nano materials: super paramagnetic (SPIONs)	Optical: Fluorescence	Water	*E. coli* cells		Absolute	1 × 10^2^ CFU	Pandit et al., 2022
Quenching of AuNCs by Cu2+ and copper-homeostasis	Bacteria	Nano composites: photostable blue carbon dots, Silica and target sensitive gold nano clusters (BCD@SiO2@AuNC)	Optical: Fluorescence	Swimming pool water, Egg shell and Cabbage	Gram negative	1.5 h	Relative	10^3^–10^7^ CFU/ml	Fu et al., 2022
QscR quorum sensing [an enhanced green fluorescent protein (EGFP)]	Pathogens: bacteria (whole cell)	Fiber: paper-based assay by immobilizing lycopene-based whole-cell	Optical: fluorescence	Water	Pseudomonas aeruginosa and Burkholderia pseudomallei	3–8 h	Absolute	2 × 10^–9^ M	Wu et al., 2021
Electrochemical impedance spectrometer (EIS)	Bacteria	Cationic polymers: surface coatable fluorescent carbon dots	Electrochemical and Luminescent	Water	*E. coli* and *Staphylococcus aureus*	Real	Absolute	70 and 131 CFU/ml	Robby et al., 2021
Thiolated-oligonucleotides capturing probes	Protozoa	Nano materials: Gold nano particles	Optical: colorimetry	Water	*Cryptosporidium*	30 min	Absolute	5 μM	Luka et al., 2021
Electrolyte-gated field-effect transistors (EG-FETs)	Protein	Inkjet-printed polymer-wrapped monochiral single-walled carbon nanotubes (s-SWCNTs)	Electrochemical: potentiometric	Aqueous environment	Molecular and pathogenic detection	1–4 h	Relative	1.47 nM	Molazemhosseini et al., 2021
Screen-printed carbon electrode (SPCE)	Bacteria: antibodies	Anti-body-decorated gold nanorods (Au-NRs-Avidin-Ab-E)	Electrochemical: impedance	Water	Escherichia coli	30 min	Relative	0.37 CFU/ml	Panhwar et al., 2021
Gold, platinum wire, and Ag/AgCl electrode	Bacteria: cDNA	Beads: magnetic beads	Electrochemical signals: potentiometric	Tap and lake water and Honey	*Staphylococcus aureus*	2 h	Absolute	8 CFU/ml	Cai et al., 2021
Plate reader (iD3, molecular devices)	Immuno-sensing, antibody based	CRISPR/Cas12a reaction solution	Optical: fluorescence	Waste water	*Cryptosporidium parvum* oocysts	∼ 2.5 h	Absolute	10 oocysts/ml	Li et al., 2021
GCE/TEMPO –ZnPc	Bacteria	Coating of zinc phthalocyanine bearing stabile TEMPO radical groups (TEMPO-ZnPc) on a glassy carbon electrode (GCE)	Electrochemical: amperometric	Milk and water	*Pseudomonas aeruginosa* [acyl homoserine lactones (AHLs)]	5 s	Absolute	1.8 × 10^–6^ mol/dm^3^	Özcan et al., 2020
Micro electrochemical sensor	Bacteria	Micro Gold electrodes T4B-MES/Au	Electrochemical: voltametric (DPV: Differential Pulse Voltammetry)	Water	*Escherichia coli B*	20 min	Absolute	14+5 CFU/ml	Xu et al., 2020
Differential pulse voltammetry/DNA three-dimensional micro total analysis system (μTAS 3D)	Protozoa	Circular gold coated area, polyimide (PI) sheet and screen printed electrode	Electrochemical: impedance	Water	*Cryptosporidium*	60 min	Relative	1.8 ng/ml	Ilkhani et al., 2019
Aptamer NanoZyme sensor	Bacteria	Nano particles: gold nanoparticles (GNPs) and *Pseudomonas aeruginosa–*specific aptamer (F23)	Optical: Colometry; electrochemical: amperometric	Water	*Pseudomonas aeruginosa*	10 min	Absolute	∼ 60 CFU/ml	Das et al., 2019
Mass sensitive	Bacterial Pathogen	Polyaniline coated filter paper functionalized with a homo-bi functional crosslinker, glutaraldehyde and 2 gold electrodes with filter paper using conductive silver paste	Electrochemical: impedance (Frequency response analyzer)	Tap and gray water	*Total bacterial load*	20 min	Absolute	500–1000 CFU/ml	Mondal et al., 2019
Impedimetric paper-based biosensor	Bacteriophage	Fiber: paper	Electrochemical: impedance	Synthetic wastewater	Cultures from sewage sludge	5 days	Relative	1.9 × 10^3^ CFU/ml	Rengaraj et al., 2018
Real-time amperometric immunoassay amplified by nanomaterial	Bacteriophage	Nano materials	Electrochemical: amperometric	Water	*E. coli*	3.5 days	Relative	50 CFU/ml	Altintas et al., 2018
Amino Acid based	Bacteria	Microcontact imprinting	Electrochemical	River water	*E. coli*	Not specified	Absolute	70 CFU/ml	Idil et al., 2017
Phage-mediated separation with quantitative PCR detection	Bacterial DNA	Beads: magnetic beads	Amplification of target DNA: qPCR	Agricultural water and city water	*E. coli* O157:H7	2 h	Relative	10^2^ CFU/ml	Wang et al., 2016

Biosensors are more precise and accurate for detecting pathogens in water and environmental samples than conventional methods. These sensing techniques have grown to be more adaptable, influential and dynamic with the aid of nanosized materials. The transduction process has significantly improved using various nanomaterials, enabling faster detection, higher sensitivity, shorter response time, and reproducibility. However, there are still some challenges that need to be overcome. For instance, ensuring quality, regulatory compliances, commercialization cost (limited applications due to high cost), production of heat-resistant biosensors (Chocarro-Ruiz et al., 2017), further improvement in terms of reusability, stability and reproducibility (Li T. et al., 2020), and expansion of range and ability for detecting various pathogens in water at the same time (Herrera-Domínguez et al., 2023). The future perspective is developing microfluid, lab-on-chip and clustered regularly interspaced short palindromic repeats (CRISPR-Cas), small, portable and automated devices with digital electrodes along with nanotechnologies with various transducers for enhancing pathogenic detections in water (Kumar et al., 2018; Wu et al., 2021). Some biosensor tools can also be developed with a thorough quantitative microbial risk assessment to prevent future outbreaks.

## 5 Innovative applications and case studies

### 5.1 Case studies on waterborne pathogen detection technology

Recent innovative case studies elucidate state-of-the-art technology used for the detection of waterborne pathogens has improved the speed, portability, cost-effectiveness and accuracy of identifying harmful pathogens in water sources (Table 3).

**TABLE 3:** Case studies of waterborne pathogenic bacteria detection methods.

Detection methods	Quantification	Target pathogen	Portable	Sample source	Limit of detection	Time-to-result	Cost	References
qPCR assay: miniature speaker-sized Q qPCR instrument	Absolute	*E. coli* (rod A)	Yes	Catchment water	10 genes/μL template	<3 h	Equipment: £9980 £20 per assay to test 1000 samples	Zan et al., 2022
PCR coupled with lateral-flow bioassay (RPA-LFA)	Absolute	*E. coli* O157:H	Yes	Drinking water, apple juice, and milk	10 fg DNA and 102 CFU/mL for the fliC gene target	8 min	NA	Rani et al., 2021
Microfluidic portable system	Absolute	*E*. *faecalis*	Yes	River water	10^1^ to 10^3^ CFU/mL	Multiple sample detection < 1 h, <5 min	NA	Gowda et al., 2022
Most probable number-loop-mediated isothermal amplification (MPN-LAMP) approach on a polymethyl methacrylate (PMMA) microchip	Absolute	*E. coli* and *Enterococcus* spp.	Yes	Raw sewage, beach, agricultural runoff, and reservoir water	4 copies/well	<2 h	NA	Fu et al., 2021
PMA-qPCR	Absolute	*E. coli*	No	Water treatment plant	10^1^ genomic units/reaction	N/A	N/A	Kibbee and Örmeci, 2017
PMA-qPCR	Absolute	Viable but non-culturable opportunistic pathogens	No	Drinking water treatment plant	10^0^–10^1^ gene copies/reaction	N/A	N/A	Guo et al., 2021

PCR, recombinase polymerase amplification; PMA, propidium monoazide (PMA); NA, not available.

The portability of waterborne pathogen detection methodologies is desirable for onsite and low-resource settings, enabling efficient and accessible diagnostic capabilities even in challenging environments. Zan et al. (2022) demonstrated rapid water quality testing using an onsite qPCR assay in a mobile laboratory. They quantified HF183 marker genes for human host-associated Bacteroides in river water within 3 h of sampling. The mobile laboratory was then deployed for fecal pollution source tracking in an urban catchment. Notably, the use of portable equipment items resulted in an 87% reduction in weight and a 53% reduction in costs compared to conventional laboratory equivalents. As the on-site qPCR assay equipment could easily be packed into a suitcase, the method is easily deployable for remote settings or abroad. Additionally, the study of Fu et al. (2021) shared insights into rapid and low-cost methods for waterborne pathogen detection and monitoring, where most probable number-loop-mediated isothermal amplification (MPN-LAMP) assay with polymethyl methacrylate PMMA-based microchips was applied for quick quantification of fecal indicator in water bodies.

Recently, a study demonstrated the development of a portable pathogen analysis system capable of detecting bacteria in water and suitable for point-of-sample collection. The system utilizes a centrifugal microfluidic platform to integrate bacterial cell lysis, nucleic acid extraction and reagent mixing with a droplet digital loop-mediated isothermal amplification assay for bacteria quantification onto a single centrifugal disc. The prototype system successfully detects *Enterococcus faecalis* in water samples within an hour, requiring less than 5 min of hands-on time (Gowda et al., 2022).

The presence of viable but non-culturable (VBNC) bacteria has raised substantial concerns as they cannot undergo division on typical culture media, but they retain some level of metabolic activity and maintain their infectious potential (Dietersdorfer et al., 2018). Under favorable conditions, these bacteria can be resuscitated (Chen et al., 2018). Consequently, conventional culture-based methods may significantly underestimate the prevalence of VBNC bacteria, which can pose a potential threat. To address the need for differentiating between viable and non-viable microorganisms, viability PCR was developed. By pre-treating the sample with specific intercalating photoreactive dyes such as propidium monoazide and ethidium monoazide, DNA from live cells can be selectively detected by PCR (Deshmukh et al., 2016; Zhong and Zhao, 2018). Guo et al. (2021) combined culture-dependent methods and quantitative PCR with propidium monoazide dye to assess cellular viability in a drinking water treatment facility. The study also developed a method to quantify viable pathogens by establishing a correlation between specific gene copies and viable cell numbers. In addition, Kibbee and Örmeci (2017) developed a highly sensitive and accurate propidium monoazide-quantitative polymerase chain reaction (PMA-qPCR) assay to quantify viable but non-culturable *E. coli* in secondary wastewater effluent after chlorine disinfection.

In line with the goal of rapid, portable, sensitive and specific pathogen detection, Rani et al. (2021) developed a method that comprised recombinase polymerase amplification coupled with lateral-flow bioassay (RPA-LFA) to detect *E. coli* O157:H7 in drinking water, apple juice and milk. Recombinase polymerase amplification (RPA) is a molecular biology technique used to amplify DNA quickly at a low temperature. Although it amplifies DNA like PCR, it uses different enzymes and reaction conditions. RPA uses recombinase enzymes to facilitate strand exchange and polymerase enzymes to extend the primers, while PCR uses DNA polymerase to extend the primers. The RPA-LFA method can be completed within 8 min at temperatures between 37 and 42°C, requiring minimal handling and simple equipment. The target amplification threshold is achieved within 5–30 min of incubation. It shows great promise as a rapid and effective alternative to conventional methods for monitoring *E. coli* O157:H7 in food and water.

### 5.2 Clustered regularly interspaced short palindromic repeats (CRISPR-Cas) for pathogenic bacteria detection

Clustered Regularly Interspaced Short Palindromic Repeats (CRISPR-Cas) is an emerging molecular biosensing tool for the detection of DNA, RNA and other biomarkers. The tool has unique sensing properties like elevated recognition specificity, sensitivity, single base resolutions, reaction at room temperature and targeted induced (Yin et al., 2021). Along with molecular sensing, the tool can also detect non-molecular or non-nucleic acid targets such as ligands, proteins, allergens, exotoxins, endotoxins, DNAzyme. Off-target effect or wrong cutting of DNA, sequence limitation, sample pretreatments, multiplexed detection, dynamic range and sophisticated instrument dependence are some associated limitations and challenges for the tool (Li et al., 2022b). Biosensors based on CRISPR-Cas have the potential for florescence, colorimetric, electrochemical, qualitatively and quantitatively biosensing of infectious pathogens, and also sense targeted specific nucleic acids and minor alteration in sequences with greater accuracy (Mao et al., 2022). Rapid and point-of-need multifaceted advantage of CRISPR-cas12a and surface enhanced Raman scattering along with recombinase polymerase amplification with integrated microfluidic paper-based analytical device were designed for super-sensitive detection of *Salmonella typhimurium*. The detection limit was 3–4 CFU/ml, detection range of 1 to 10^8^ CFU/ml in 45 min (Zhuang et al., 2022). Biosensor for ultrasensitive detection of specific *inv*A-sequence of *Salmonella typhimurium* was designed with CRISPR-Cas 12a and gold nanoparticles (Ma et al., 2021), silver nanocluster termed SCENT-Cas (Silver nanocluster Empowered Nucleic acids Test using CRISPR-Cas 12a) (Ma et al., 2023), and SCOUT-dCas9 (ultra-sensitive, cross-validating, on-site and dual-mode test using CRISPR/dCas9) (Li et al., 2023b). CRSIPR-Cas 12a and gold nanoparticles detection limit and dynamic range is 1 CFU/ml and 10^0^ to 10^8^ CFU/ml, respectively, SCENT-Cas has 1 CFU/ml and 1 to 10^8^ CFU/ml, and SCOUT-dCas9 has 1 CFU/ml and 1 to 10^9^ CFU/ml. The CHANCE (Cas 12a-HCR evANescent wave fluorescenCE) was used by Song et al. (2023) for rapid and sensitive detection of *E. coli* in actual environmental water sample using specific crRNA with *rfb*E gene of *E. coli* O157:H7 as target. The detection limit was 17.4 CFU/ml with a concentration range from 10 to 10^8^ CFU/ml in 50 min.

Similarly, Argonaute has also recently been used as a biosensing tool for the detection of nucleic acid. Biosensors mediated with Argonaute have higher specificity and recognition ability for nucleic acids. Li et al. (2023a) developed a versatile NOTE-ago (Novel and One-step cleavage method based on Argonaute by integrating Tag-specific primer extension and Exonuclease I) for the detection of pathogens (*Salmonella typhi* and *Staphylococcus aureus*). The NOTE-ago based biosensor can detect 1 CFU/ml with a range of 1 to 10^8^ CFU/ml in real samples. CRISPR-Cas can widely be used for genome editing, targeting DNA and RNA, and can be programmed to target specific DNA sequences as well as offers multiplex targeting. While, Argonaute is extensively used and studied for gene regulation, RNA interferences and targeting single RNA molecule. Overall, CRISPR/Cas and Argonaute differ in their mechanisms of action, target specificity and applications in the nucleic acid tests (Li et al., 2022a).

### 5.3 Artificial intelligence (AI) integrated technologies for waterborne bacteria detection

Researchers have integrated artificial intelligence (AI) with microbial image analysis in numerous studies to tackle the issues in human sensing data assessment. The use of machine learning (ML) and deep learning (DL) for microbial counting have been increasing steadily since 2015, ascribed to the immense growth and development of DL algorithm, contributing to more accurate image segmentation (Zhang et al., 2022). In addition, ML has been proven effective in handling big sensing data from complex samples, as well as producing reasonable results from noisy and low-resolution sensing data (Cui et al., 2020).

Another compelling development is a computational live bacteria detection system that combines time-lapse coherent imaging, and DL for rapid detection and classification of bacterial growth (Wang et al., 2020). This test system could swiftly identify and classify *E. coli*, *Klebsiella aerogenes*, and *Klebsiella pneumonia* with 90 and 80% accuracy for detection and classification, respectively. This system reduced the detection time significantly by more than 12 h compared to the EPA-approved gold standard methods and achieved a limit of detection (LOD) of ∼1 CFU/L in less than 9 h of total test time. The system is highly cost-effective and suitable for integrating agar plates with existing bacteria detection methods.

Smartphones have emerged as a promising tool for on-site assays for molecular and biosensing approaches for pathogenic bacteria detection. Smartphones come into sight to streamline the process as they simplify the device, enhance portability and maintain essay sensitivity. A study employing supervised ML (support vector machine) and smartphone-based paper microfluidic analysis achieved an impressive accuracy of 93.3% in bacterial species classification (Kim et al., 2021). Peptide-conjugated particles and bacterial suspensions are loaded onto paper microfluidic chips, and the flow velocity data generates a unique fingerprinting profile for each bacterial species.

A recent AI biosensing framework for rapid pathogen detection in agricultural water and liquid food was developed using a DL model to identify and quantify target bacteria based on their microscopic patterns generated by specific bacteriophage interactions (Yi et al., 2023). This AI-enabled biosensing achieved rapid prediction within 5.5 h with accuracy ranging from 80 to 100 % on real-world water samples. The framework showcases the potential for AI-based rapid pathogen detection in agricultural water, significantly reducing the need for extensive human and laboratory effort. It also demonstrates its applicability to real-world water samples with complex backgrounds. The integration of AI technology into pathogenic bacteria detection and their classification are summarized in Table 4.

**TABLE 4:** Integration of artificial intelligence (AI) technology into pathogenic bacteria detection techniques and classification.

Target microorganisms	Matrix	Sensing mechanism	AI algorithm	Accuracy	References
*E. coli*	Agriculture water	Microscopic imaging	Faster-R convolutional neural network	80–100%	Yi et al., 2023
*E. coli*	Groundwater	Colorimetry	Convolutional neural network	96%	Khan et al., 2021
*E. coli*, *Staphylococcus aureus*, *Salmonella Typhimurium*, *Enterococcus faecium, and Pseudomonas aeruginosa.*	Pond water	Microfluidic analysis using bacteria-particle aggregation	Support vector machine	93.30%	Kim et al., 2021
*E. coli*, *Klebsiella aerogenes*, and *Klebsiella pneumonia*	Prepared reagent grade water and culture media	Time-lapse imaging	Deep neural network	89–98%	Wang et al., 2020

Incorporating AI, ML, and DL into waterborne pathogens detection technologies can be helpful for identifying unusual patterns that could indicate contamination. This approach allows for early interventions that prevent potential issues from escalating into public health crises.

### 5.4 Development consideration for waterborne pathogenic bacteria detection technologies

When developing waterborne pathogenic bacteria detection technologies, it is crucial to align with global standards like UNICEF TPP, adhere to the ASSURED and REASSURED criteria, incorporate circular economy principles, and draw insights from case studies and past scientific research to ensure affordability, accessibility, accuracy and environmental sustainability. The ASSURED guidelines proposed by the WHO (2003) are the most critical consideration. These guidelines emphasize the importance of affordability, sensitivity, specificity, rapidity and robustness, equipment-free operation and deliverability (Kettler et al., 2004). Even though these criteria do not directly apply to waterborne detection, they were widely accepted and served as practical guidelines for innovation in pathogen detection technologies at all levels, including in resource-limited settings.

After over a decade, two additional criteria have been added to ASSURED for consideration as “REASSURED” (Mazur et al., 2023). The updated criteria now emphasize the importance of real-time connectivity and ease of specimen collection. These additions reflect the evolving needs and technological developments in the field, ensuring that the detection methods align with the latest requirements and facilitate efficient and convenient testing processes. The World Health Organization Special Program for Research and Training in Tropical Diseases (WHO/TDR) introduced a set of criteria in 2003 to establish an ideal test suitable for all levels of the healthcare system in developing countries. This comprehensive framework, ASSURED, was developed to guide treatment and clinical management decisions in infectious diseases. Since then, it has become widely accepted as the standard benchmark for an ideal point-of-care (POC) test, ensuring its practicality and applicability in resource-limited settings.

Furthermore, it is essential to consider product design and life cycles based on key documents such as the WHO policy in 2025, national policy targets and relevant case studies. By incorporating these considerations, the technology transfer process can be guided by best practices and aligned with the latest industry standards and scientific advancements. Technology transfer should also include the circular economy framework, which aims to use the end product as the manufacturing source to minimize and recycle waste as much as possible. A thorough consideration emphasizes minimizing waste in product manufacturing and promoting the use of recycled materials to reduce environmental impact, in line with the United Nations Sustainable Development Goal on responsible consumption and production (SDG 12). Due to its adverse health effects, recent changes include eliminating bisphenol A (BPA) from polycarbonate plastics and food products.

However, concerns have arisen regarding plastic waste from non-recyclable test cassettes used in COVID rapid tests, similar concern should also be considered for waterborne pathogen detection test kit. While the WHO and UNICEF recommend mandatory incineration with energy recovery for cassettes used in infectious diseases, low-resource regions often resort to landfilling, despite its adverse environmental effects. When designing diagnostic tests, it is crucial to consider a cost-to-benefit ratio, where the acceptable cost depends on the expected benefit. Additionally, marketing strategies and the availability of materials in the deployment region should be considered, including the use of readily available materials.

The review also conducted an analysis of pairwise comparison matrices to assess seven criteria (affordability, sensitivity, specificity, user-friendliness, rapidness, equipment-free, and deliverable to end-users) of waterborne detection technology according to ASSURED proposed by WHO. This review systematically evaluated and ranked each characteristic based on its relative importance and performance compared to others (Table 5 and Supplementary Table 4). Meanwhile, waterborne detection method selection considerations are summarized in Supplementary Table 5. This rigorous approach provides a clear overview of the strengths and weaknesses of different technologies and offers valuable insights for selecting the most suitable methods for waterborne pathogen detection.

**TABLE 5:** Comparative score for various detection techniques according to WHO’s ASSURED criteria.

Method	Comparative score	
Culture plating	Affordability	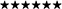
	Sensitivity	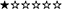
	Specificity	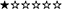
	User-friendliness	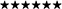
	Rapidness	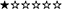
	Equipment-free	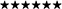
	Deliverable	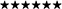
Quantitative real-time PCR (qPCR)	Affordability	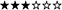
	Sensitivity	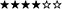
	Specificity	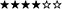
	User-friendliness	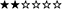
	Rapidness	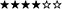
	Equipment-free	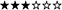
	Deliverable	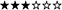
Loop-mediated isothermal amplification (LAMP)	Affordability	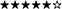
	Sensitivity	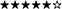
	Specificity	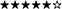
	User-friendliness	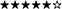
	Rapidness	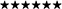
	Equipment-free	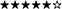
	Deliverable	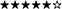
Enzyme-linked immunosorbent assay (ELISA)	Affordability	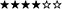
	Sensitivity	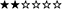
	Specificity	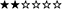
	User-friendliness	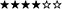
	Rapidness	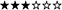
	Equipment-free	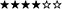
	Deliverable	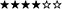
Flow cytometry (FCM)	Affordability	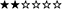
	Sensitivity	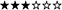
	Specificity	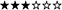
	User-friendliness	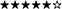
	Rapidness	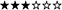
	Equipment-free	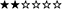
	Deliverable	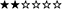
Next-generation sequencing (NGS)	Affordability	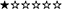
	Sensitivity	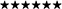
	Specificity	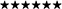
	User-friendliness	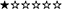
	Rapidness	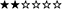
	Equipment-free	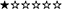
	Deliverable	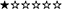

The comparative score (WHO’s ASSURED criteria) for detection techniques (culture plating, qPCR, LAMP, ELISA, FCM, and NGS) were obtained from pairwise comparison matrices (Supplementary Table 4) are based on informed judgment and available literature rather than quantitative measure.

## 6 Gaps in current knowledge, challenges, and future perspective

Substantial progress has been made in the development of detection technologies for waterborne pathogenic bacteria, but there remain significant challenges related to fulfilling the WHO’s ASSURED criteria, namely, affordable, sensitive, specific, user-friendly, rapid and robust, equipment-free, and deliverable to end-users. The respective challenges are discussed as follows.

### 6.1 Challenges

Waterborne pathogenic bacteria detection methods have undergone significant evolution over the years, leading to an array of sophisticated techniques. However, challenges persist spanning from fundamental sensitivity concerns to complex issues posed by new technologies.

Sample complexity is a significant challenge for pathogen detection techniques as natural water sources usually present a matrix rich in microorganisms, suspended particles and organic matter. This complexity can introduce interference in detection methods. For instance, readings from techniques dependent on colorimetric or fluorescence outputs can be skewed by naturally occurring elements. Molecular-based methods are notable for high precision but also encounter obstacles related to the purity of DNA or RNA samples. Contaminants from sources like humic acids and phenolic compounds can inhibit enzymatic reactions. Under these circumstances, traditional extraction methods struggle to yield high-quality nucleic acids from complex samples, emphasizing the need for specialized extraction approaches. Furthermore, the precise quantification of pathogens is fraught with challenges, especially with potential interferences affecting the concentration determination. To counter these challenges, pre-treatment steps are integrated, but these may introduce more complexity and potential errors. Additionally, environmental factors such as temperature and turbidity further complicate the detection of pathogenic bacteria (Weller et al., 2020). Temperature variability can influence the viability and detectability of pathogens, posing a risk of inconsistent results. while turbidity challenges optical detection and introduces potential contaminants.

The sensitivity of detection techniques is another hurdle. The low concentrations of pathogens in water samples can make detection challenging. High sensitivity is important but certain methods like immunoassays risk producing false positives due to cross-reactivity with unrelated substances. Meanwhile, another challenge arises from the very nature of bacteria. Certain bacteria can transition into a viable but non-culturable (VBNC) state, rendering them undetectable by conventional methods, yet capable of becoming active under favorable conditions (Chen et al., 2018). This VBNC state underscores the limitations of several detection techniques that may overlook these dormant bacteria.

Cost is another significant impediment. Advanced detection methods offer accuracy, but come with substantial costs. The expenses can range from the purchase of sophisticated equipment to the procurement of specialized reagents and training and can be prohibitive, particularly for facilities in developing regions or areas with limited resources. Coupled with the economic challenges is the need for timely detection. Traditional methods are more affordable, however, they are also time-consuming, which may potentially lead to delayed interventions and increased health risks. Thus, the ongoing quest in waterborne pathogen detection is to develop techniques that are both economically feasible and timely, ensuring that threats are swiftly identified and mitigated.

In essence, the challenges of the detection technologies also illuminate avenues for further research and development. Continued research efforts and innovations should focus on developing methods that balance the trade-offs between sensitivity, specificity, cost and timeliness.

### 6.2 Future perspectives

Waterborne pathogenic bacteria pose significant threats to public health and the environment and require continued development of detection technologies to ensure timely and accurate identification. As we explore the prospects of waterborne bacteria detection, it becomes evident that multidisciplinary approaches and innovative technologies will play essential roles in addressing current knowledge gaps and expanding the boundaries of detection capabilities. Several areas are worth emphasizing for future direction, such as AI, nanotechnology in biosensors as well as lab-on-chip technologies.

#### 6.2.1 Integration of artificial intelligence (AI)

The integration of artificial AI has the potential to transform waterborne bacteria detection (Yi et al., 2023). Machine learning and deep learning algorithms can analyze vast datasets, recognize patterns, and learn from past data to improve the accuracy and efficiency of detection (Cui et al., 2020). By leveraging AI, researchers can develop predictive models that identify potential outbreaks, evaluate water quality trends, and optimize real-time monitoring strategies. Furthermore, AI-driven data processing can enable rapid decision-making, making it an invaluable tool for public health officials in mitigating waterborne disease outbreaks. Infrastructure needs, such as computational power and continuous model training are essential components for this integration. As the technology evolves, AI’s role in detecting waterborne bacteria is anticipated to grow, and it requires a balanced approach to harness its potential and address challenges.

#### 6.2.2 Nanotechnology-enhanced biosensors with signal amplification

The integration of nanotechnology into biosensors for detection of waterborne pathogenic bacteria is growing. It leverages the physicochemical properties of nanomaterials to enhance signal transduction and consequently improve both sensitivity and specificity. Nanomaterials, particularly those with a three-dimensional structure and tailored morphology, are crucial in enhancing electrochemical detection. When electrodes modified with these nanomaterials are paired with electrochemical labels, it propels advancements in practical applications and commercial viability of electrochemical detection techniques. Additionally, it creates versatile and portable detection devices that can be easily integrated with mobile communication systems that leads to more efficient and affordable solutions for the concurrent detection of pathogens (Bai et al., 2022).

The advancement in nanostructured detection techniques delivers consistent and reproducible outcomes in a reduced detection time, coupled with a lower limit of detection and such advancements are vital in rapid detection of *E. coli* contamination in food and water (Zhou et al., 2023). Additionally, the miniaturization potential of nanotechnology facilitates the development of portable and field-ready devices, particularly beneficial in remote environments or emergent situations. In addition, the developments in biosensor signal amplification employ strategies like nanomaterial-based enhancers and enzymatic cascades can further heighten sensitivity of trace bacterial contaminants detection, hence minimize false-negative outcomes. Future investigations will merge these innovations and further explore diverse nanomaterials and amplification mechanisms to boost the detection capability and reliability of biosensors.

#### 6.2.3 Lab-on-chip and microfluidic technologies for waterborne pathogenic bacteria detection

A lab-on-chip (LOC) device is a miniaturized laboratory system that integrates several functions on a single chip, which is typically small in size (only millimeters to a few square centimeters). The integration of microfluidics with LOC technologies marks an innovatory move in waterborne bacteria detection. Compared to conventional methods, LOC offer unique features such as compact, automation capabilities, and proficiency in executing multiplexed analyses using tiny sample volumes. This innovative design streamlines sample processing, significantly reduce the time for analysis and enhance the detection efficiency (Gagliardi et al., 2023). With the capacity to combine multiple detection techniques such as PCR, immunoassays, and nucleic acid sequencing within one unified platform, LOC technologies empower users to conduct numerous assessments of water quality almost instantly.

One of the advantages of LOC devices is the potential for high-throughput analysis. The inherent portability also makes it ideal for consistent monitoring in varied settings, from well-equipped labs to more challenging environments like remote areas. However, LOC devices have limitation in standardization across different applications and ensuring consistent sensitivity. As the technology advances, the future of LOC technologies will be more promising with the anticipated integration of AI and data analytics.

#### 6.2.4 Metagenomic approaches

Metagenomic techniques such as next-generation sequencing (NGS) has emerged as powerful tools for exploring microbial diversity in water environments. NGS allows simultaneous analysis of entire microbial communities and provides a more comprehensive understanding of the bacterial composition as well as the presence of potential pathogens (Ko et al., 2022). In contrast with traditional microbial detection methods, which often focus on a limited set of known pathogens, NGS stands out due to its capacity for a broader, simultaneous analysis. Such comprehensive insights are crucial, especially in the context of a rapidly changing environment where new pathogenic threats can emerge.

However, metagenomic approaches come with shortcomings. One pertinent issue is the enormous data sets NGS produces, which can be overwhelming to interpret. The intricacy of microbial ecosystems with multitude interactions also requires sophisticated bioinformatic tools for accurate analysis. Historical instances, such as the detection of previously unknown pathogens in contaminated water sources underscore the transformative potential of NGS.

#### 6.2.5 Hybrid detection systems

The potential of hybrid detection systems in waterborne bacterial detection lies in the integration of diverse techniques, where each technique offers its unique strength. For example, PCR is renowned for its precise amplification and specificity, complements the real-time detection and portability of biosensors, while metagenomics offers a panoramic view of microbial communities. This incorporation exploits on individual advantages and offsets inherent limitations, such as PCR’s sensitivity to inhibitors or the data-intensive nature of metagenomics. Emerging prototype systems validate the feasibility of this integrated approach, emphasizing its potential to drastically reduce false positives and negatives. However, challenges like reconciling method discrepancies, achieving uniform sample handling, and mitigating costs persist. To attain greater sensitivity, the CRISPR/Cas system is often paired with the PCR and other isothermal nucleic acid amplification methods such as nucleic acid sequence-based amplification (NASBA), rolling circle amplification (RCA), strand displacement amplification (SDA), loop-mediated isothermal amplification (LAMP), recombinase polymerase amplification (RPA), and exponential amplification reaction (EXPAR). By integrating CRISPR/Cas with these cutting-edge amplification techniques, the emergence of innovative optical and electrochemical biosensing devices is being fostered (Chakraborty et al., 2022).

The future of waterborne bacteria detection hinges on leveraging cutting-edge technologies and embracing interdisciplinary collaboration. Advancements in AI, nanotechnology, microfluidics, metagenomics, and biosensors offer a promising trajectory to fill the existing knowledge gaps and improve the accuracy, sensitivity and speed of waterborne bacteria detection. As researchers work toward developing innovative, portable and reliable detection methodologies so society is better equipped to safeguard public health, protect water resources and effectively respond to waterborne disease outbreaks.

## 7 Conclusion

In conclusion, while a diverse range of techniques for the detection of waterborne pathogenic bacteria exists, each has its own advantages and limitations. Conventional techniques like cultured-based methods and PCR techniques have laid the groundwork, but emerging technologies such as digital and multiplex PCR, and NGS, are pushing the boundaries of what is possible in this field. The evolving domain of biosensors, particularly those based on nanomaterials, is paving the way for a more sensitive, specific, and rapid detection of waterborne bacteria. Despite these advancements, there remain gaps in our knowledge, particularly in the context of practical implementation and large-scale usability. As the field continues to evolve, future research should focus on the development of robust, cost-effective, and user-friendly techniques that can be widely adopted for routine monitoring and rapid detection of waterborne bacteria, thereby safeguarding global water supplies and public health.

## Author contributions

Y-SO: Conceptualization, Formal analysis, Funding acquisition, Methodology, Project administration, Validation, Visualization, Writing – original draft, Writing – review and editing. MA: Formal analysis, Validation, Visualization, Writing – original draft, Writing – review and editing, Data curation, Investigation. MD: Conceptualization, Supervision, Validation, Writing – review and editing. LL: Conceptualization, Supervision, Validation, Writing – review and editing. KS: Funding acquisition, Supervision, Validation, Writing – review and editing, Conceptualization.
