# Supplementary material for: Waterborne pathogens detection technologies: advances, challenges, and future perspectives
Source: Front Microbiol. 2023 Nov 23;14:1286923. doi: 10.3389/fmicb.2023.1286923 (PMC10708915; doi:10.3389/fmicb.2023.1286923)
Supplement: Supplementary file 1 [file Table_1.docx]

Supplementary Material

**List of Supplementary Table**

**Supplementary Table 1** Comparison studies of pathogenic bacteria detection with NGS method

**Supplementary Table 2** Early development of biosensor

**Supplementary Table 3** Bioreceptors in biosensors for recognition of pathogens

**Supplementary Table 4** Pairwise comparison matrices for comparative scores of various pathogenic bacteria detection techniques

**Supplementary Table 5** Summary of waterborne detection method selection considerations

**Supplementary Table 1.** Comparison studies of pathogenic bacteria detection with NGS method

| **Detection Method** | **Annotation database** | **Type of bacteria** | **Sample matrices** | **Location** | **Reference** |
| --- | --- | --- | --- | --- | --- |
| Illumina MiSeq sequencing | SILVA 16S rRNA database and RDP Classiﬁer (http://rdp.cme.msu.edu/) | *Proteobacteria, Bacteroidetes, Acidobacteria,* and *Chloroflexi*. | River | Taihu, China | (Vadde et al., 2019) |
| Illumina MiSeq | Greengenes ZOTUs | *Proteobacteria, betaproteobacteria, Gammaproteobacteria, Arcobacter venerupis, Laribacter hongkongensis* and *Neisseria canis* | Wastewater treatment | Northwest Western Australia | (Greay et al., 2019) |
| De novo sequencing | 16S rRNA – MEGA v7.0.26NCBI, LPSN, Prokka | *Legionella pneumophila* | Man-made water systems | 15 countries | (Svetlicic et al., 2023) |
| Illumina HiSeq | 16S rRNA gene (approximately 466 bp) | ﻿*E. coli*, *Entero- coccus faecalis*, *Campylobacter jejuni, Arcobacter cryaerophilus, Acinetobacter johnsonii, Acinetobacter lwoffii* and Aeromonas spp. | Rivers and sewage plant | Changzhou City of Yangtze River Delta | (Cui et al., 2019) |
| Illumina Miseq | NA | *Legionella. pneumophila SG1 ST138* | Bath facility | Japan | (Nakanishi et al., 2023) |

NA: Not Available

**Supplementary Table 2.** Early development of biosensors

| **Biosensor** | **Year** | **Reference** |
| --- | --- | --- |
| Ion-sensitive field-effect transistor (ISFET) | 1970 | (Bergveld, 1970) |
| Fibre-optic biosensor for carbon dioxide and oxygen detection | 1975 | (Vestergaard et al., 2015) |
| Biosensor for glucose detection | 1975 | (Yoo and Lee, 2010) |
| First microbe-based immunosensor | 1975 | (Suzuki et al., 1975) |
| Fibre-optic biosensor for glucose detection | 1982 | (Schultz, 1982) |
| Surface plasmon resonance (SPR) immunosensor | 1983 | (Liedberg et al., 1983) |
| Amperometric biosensor: ferrocene used with glucose oxidase for glucose detection | 1984 | (Cass et al., 1984) |
| SPR-based biosensor | 1990 | (Vestergaard et al., 2015) |
| Handheld blood biosensor by i-STAT | 1992 | (Vestergaard et al., 2015) |
| Nano biosensor | 1999 | (Poncharal et al., 1999) |

**Supplementary Table 3.** Bioreceptors in biosensors for recognitions of pathogens

| **Bioreceptor** | **Principle** | **Reference** |
| --- | --- | --- |
| Antibody bio-receptors (recombinant, polyclonal or monoclonal) | Lock and key fitting | (Li T. et al., 2020) |
| Bacteriophages | Transformation of genetic material to bacteria, Tail spike protein | (Sedki et al., 2020) |
| Enzymes | Specific binding capacity and catalytic ability | (Khan et al., 2020) |
| Nucleic acid bioreceptors | Specificity | (Quintela et al., 2019) |
| Cellular bio-receptors | Entire cell/particular cellular element | (Gupta et al., 2019) |
| Cellular bio-receptors: *Cellular systems* | Responsiveness and biochemical stimuli | (Hassan et al., 2020) |
| Cellular bio-receptors: *non-enzymatic proteins* | Channel or carrier protein | (Lobry et al., 2019) |
| Cellular bio-receptors: *Biomimetic receptors* | Molecular imprinting: artificial binding sites | (Saylan et al., 2020) |
| Cellular bio-receptors: *Aptamers* | Small oligonucleotides/peptide molecules: bind living cells, toxins, small molecules, carbohydrates, peptides, and proteins | (Li Y. et al., 2020) |

**Supplementary Table 4** Pairwise comparison matrices for comparative scores of various pathogenic bacteria detection techniques

|  |  |  | |  | |  | |  |  | |  |  | |  | |  |  |  |
| --- | --- | --- | --- | --- | --- | --- | --- | --- | --- | --- | --- | --- | --- | --- | --- | --- | --- | --- |
| **Affordability** | **Culture** | | **qPCR** | | **LAMP** | | **ELISA** | | | **FCM** | | **NGS** |  | | **Technique** | | **Score** |  |
| **Culture** | + | | + | | + | | + | | | + | | + |  | | Culture | | 6 |  |
| **qPCR** | - | | + | | - | | - | | | + | | + |  | | LAMP | | 5 |  |
| **LAMP** | - | | + | | + | | + | | | + | | + |  | | ELISA | | 4 |  |
| **ELISA** | - | | + | | - | | + | | | + | | + |  | | qPCR | | 3 |  |
| **FCM** | - | | + | | - | | - | | | + | | - |  | | FCM | | 2 |  |
| **NGS** | - | | - | | - | | - | | | - | | + |  | | NGS | | 1 |  |
| In this matrix, "+" indicates that the technique in the row is more affordable than‘ the technique in the column, and "-" indicates it is less affordable. | | | | | | | | | | | | | | | | | | |
| **Sensitivity** | **Culture** | | **qPCR** | | **LAMP** | | **ELISA** | | | **FCM** | | **NGS** |  | | **Technique** | | **Score** |  |
| **Culture** | + | | - | | - | | - | | | - | | - |  | | NGS | | 6 |  |
| **qPCR** | + | | + | | - | | + | | | + | | - |  | | LAMP | | 5 |  |
| **LAMP** | + | | + | | + | | + | | | + | | - |  | | qPCR | | 4 |  |
| **ELISA** | + | | - | | - | | + | | | + | | - |  | | FCM | | 3 |  |
| **FCM** | + | | - | | - | | - | | | + | | - |  | | ELISA | | 2 |  |
| **NGS** | + | | + | | + | | + | | | + | | + |  | | Culture | | 1 |  |
| In this matrix, "+" indicates that the technique in the row is more sensitive than the technique in the column, and "-" indicates it is less sensitive. | | | | | | | | | | | | | | | | | | |
| **Specificity** | **Culture** | | **qPCR** | | **LAMP** | | **ELISA** | | | **FCM** | | **NGS** |  | | **Technique** | | **Score** |  |
| **Culture** | + | | - | | - | | - | | | - | | - |  | | NGS | | 6 |  |
| **qPCR** | + | | + | | - | | + | | | + | | - |  | | LAMP | | 5 |  |
| **LAMP** | + | | + | | + | | + | | | + | | - |  | | qPCR | | 4 |  |
| **ELISA** | + | | - | | - | | + | | | - | | - |  | | FCM | | 3 |  |
| **FCM** | + | | - | | - | | + | | | + | | - |  | | ELISA | | 2 |  |
| **NGS** | + | | + | | + | | + | | | + | | + |  | | Culture | | 1 |  |
| In this matrix, "+" indicates that the technique in the row is more specific than the technique in the column, and "-" indicates it is less specific. | | | | | | | | | | | | | | | | | | |
|  |  | |  | |  | |  | | |  | |  |  | |  | |  |  |
| **User-friendly** | **Culture** | | **qPCR** | | **LAMP** | | **ELISA** | | | **FCM** | | **NGS** |  | | **Technique** | | **Score** |  |
| **Culture** | + | | + | | + | | + | | | + | | + |  | | Culture | | 6 |  |
| **qPCR** | - | | + | | - | | - | | | - | | + |  | | LAMP | | 5 |  |
| **LAMP** | - | | + | | + | | + | | | + | | + |  | | ELISA | | 4 |  |
| **ELISA** | - | | + | | - | | + | | | + | | + |  | | FCM | | 3 |  |
| **FCM** | - | | + | | - | | + | | | + | | - |  | | qPCR | | 2 |  |
| **NGS** | - | | - | | - | | - | | | - | | + |  | | NGS | | 1 |  |
| In this matrix, "+" indicates that the technique in the row is more user-friendly than the technique in the column, and "-" indicates it is less user-friendly. | | | | | | | | | | | | | | | | | | |
| **Rapid** | **Culture** | | **qPCR** | | **LAMP** | | **ELISA** | | | **FCM** | | **NGS** |  | | **Technique** | | **Score** |  |
| **Culture** | + | | - | | - | | - | | | - | | - |  | | LAMP | | 6 |  |
| **qPCR** | + | | + | | - | | + | | | - | | + |  | | FCM | | 5 |  |
| **LAMP** | + | | + | | + | | + | | | + | | + |  | | qPCR | | 4 |  |
| **ELISA** | + | | - | | - | | + | | | - | | - |  | | ELISA | | 3 |  |
| **FCM** | + | | + | | - | | + | | | + | | + |  | | NGS | | 2 |  |
| **NGS** | + | | - | | - | | + | | | - | | + |  | | Culture | | 1 |  |
| In this matrix, "+" indicates that the technique in the row can provide results more quickly than the technique in the column, and "-" indicates it is slower. | | | | | | | | | | | | | | | | | | |
| **Equipment-free** | **Culture** | | **qPCR** | | **LAMP** | | **ELISA** | | | **FCM** | | **NGS** |  | | **Technique** | | **Score** |  |
| **Culture** | **+** | | **+** | | **+** | | **+** | | | **+** | | **+** |  | | Culture | | 6 |  |
| **qPCR** | **-** | | **+** | | **-** | | **-** | | | **+** | | **+** |  | | LAMP | | 5 |  |
| **LAMP** | **-** | | **+** | | **+** | | **+** | | | **+** | | **+** |  | | ELISA | | 4 |  |
| **ELISA** | **-** | | **+** | | **-** | | **+** | | | **+** | | **+** |  | | qPCR | | 3 |  |
| **FCM** | **-** | | **+** | | **-** | | **-** | | | **+** | | **-** |  | | FCM | | 2 |  |
| **NGS** | **-** | | **-** | | **-** | | **-** | | | **-** | | **+** |  | | NGS | | 1 |  |
| In this matrix, "+" indicates that the technique in the row require less specialised equipment than the technique in the column, and "-" indicates it requires more. | | | | | | | | | | | | | | | | | | |
| **Deliverable to end-users** | **Culture** | | **qPCR** | | **LAMP** | | **ELISA** | | | **FCM** | | **NGS** |  | | **Technique** | | **Score** |  |
| **Culture** | + | | + | | + | | + | | | + | | + |  | | Culture | | 6 |  |
| **qPCR** | - | | + | | - | | - | | | - | | + |  | | LAMP | | 5 |  |
| **LAMP** | - | | + | | + | | + | | | + | | + |  | | ELISA | | 4 |  |
| **ELISA** | - | | + | | - | | + | | | + | | + |  | | qPCR | | 3 |  |
| **FCM** | - | | + | | - | | - | | | + | | - |  | | FCM | | 2 |  |
| **NGS** | - | | - | | - | | - | | | - | | + |  | | NGS | | 1 |  |

In this matrix, "+" indicates that the results from the technique in the row are easier for end users to interpret and use as well as less logistical challenge than the technique in the column, and "-" indicates they are more difficult.

Generally, the '+' and '-' signs in the pairwise comparison matrices are determined by comparing the two techniques against the criterion under consideration. It represents a subjective assessment of which technique is superior based on the particular criterion. The comparative score is based on the count of ‘+’ signs in each row of each technique, and the technique with the highest count of “+” signs scores the highest, 6, while the technique with the lowest count of ‘+’ scores 1. Do note that these comparative scores from pairwise comparison matrices are based on informed judgement and available literature, rather than any quantitative measure or precise calculation.

**Supplementary Table 5** Summary of waterborne detection method selection considerations

|  | **Method selection consideration** | **Remarks** | **Detection Method** |
| --- | --- | --- | --- |
| 1 | Sensitivity and Specificity | High sensitivity to detect low concentrations of pathogens and high specificity to accurately identify the target pathogens, minimizing false-positive and false-negative results. | Biosensor > ddPCR > qPCR > microarray |
| 2 | Speed | Rapid result without extensive-labour | LAMP > Microarray > Biosensor |
| 3 | On-site capability | on-site detection methods are highly desirable, enabling real-time monitoring and immediate interventions. |  |
| 4 | Cost-effectiveness | Cost-effective, considering both the initial investment and the operational expenses. Affordability is crucial without the need for complex laboratory infrastructure or extensive sample processing., especially for resource-limited settings where waterborne diseases are prevalent. | mPCR > PCR |
| 5 | User-friendliness and Accessibility | Easy to use, requiring minimal training and expertise. It should also be accessible to a wide range of users, including field technicians, healthcare workers, and water quality professionals. | Lab-on-chip > Biosensor > |
| 6 | Compatibility with different Pathogens | Applicable to a variety of waterborne pathogens, including bacteria, viruses, and parasites. It should offer versatility in detecting different species and strains, ensuring comprehensive pathogen surveillance. | PCR based |

**References**

Bergveld, P., 1970. Development of an ion-sensitive solid-state device for neurophysiological measurements. IEEE Trans Biomed Eng 17(1), 70-71.

Cass, A.E., Davis, G., Francis, G.D., Hill, H.A.O., Aston, W.J., Higgins, I.J., Plotkin, E.V., Scott, L.D., Turner, A.P., 1984. Ferrocene-mediated enzyme electrode for amperometric determination of glucose. Anal Chem 56(4), 667-671.

Cui, Q., Huang, Y., Wang, H., and Fang, T. (2019). Diversity and abundance of bacterial pathogens in urban rivers impacted by domestic sewage. Environ. Pollut. 249, 24–35. doi: 10.1016/j.envpol.2019.02.094

Greay, T. L., Gofton, A. W., Zahedi, A., Paparini, A., Linge, K. L., Joll, C. A., et al. (2019). Evaluation of 16S next-generation sequencing of hypervariable region 4 in wastewater samples: An unsuitable approach for bacterial enteric pathogen identifification. *Sci. Total Environ.* 670, 1111–1124. doi: 10.1016/j.scitotenv.2019.03.278

Gupta, N., Renugopalakrishnan, V., Liepmann, D., Paulmurugan, R., Malhotra, B.D., 2019. Cell-based biosensors: Recent trends, challenges and future perspectives. Biosens Bioelectron 141, 111435.

Hassan, S.-u., Donia, A., Sial, U., Zhang, X. and Bokhari, H., 2020. Glycoprotein-and lectin-based approaches for detection of pathogens. Pathogens 9(9), 694.

Khan, M., Hasan, M., Hossain, S., Ahommed, M., Daizy, M., 2020. Ultrasensitive detection of pathogenic viruses with electrochemical biosensor: State of the art. Biosens Bioelectron 166, 112431.

Li, T., Jin, L., Feng, K., Yang, T., Yue, X., Wu, B., Ding, S., Liang, X., Huang, G., Zhang, J., 2020a. A novel low-field NMR biosensor based on dendritic superparamagnetic iron oxide nanoparticles for the rapid detection of Salmonella in milk. Lwt-Food Sci Technol 133, 110149.

Li, Y., Lu, C., Zhou, S., Fauconnier, M.-L., Gao, F., Fan, B., Lin, J., Wang, F., Zheng, J., 2020b. Sensitive and simultaneous detection of different pathogens by surface-enhanced Raman scattering based on aptamer and Raman reporter co-mediated gold tags. Sensors and Actuators B-Chem 317, 128182.

Liedberg, B., Nylander, C., Lunström, I., 1983. Surface plasmon resonance for gas detection and biosensing. Sensors and Actuators 4(2), 299-304.

Lobry, M., Lahem, D., Loyez, M., Debliquy, M., Chah, K., David, M., Caucheteur, C., 2019. Non-enzymatic D-glucose plasmonic optical fiber grating biosensor. Biosens Bioelectron 142, 111506.

Poncharal, P., Wang, Z., Ugarte, D., de Heer, W., 1999. A carbon nanotube field-emission electron source. Science 283, 1513-1516.

Quintela, I.A., de Los Reyes, B.G., Lin, C.-S., Wu, V.C., 2019. Simultaneous colorimetric detection of a variety of Salmonella spp. in food and environmental samples by optical biosensing using oligonucleotide-gold nanoparticles. Front Microbiol 10, 1138.

Saylan, Y., Erdem, Ö., Inci, F. and Denizli, A., 2020. Advances in biomimetic systems for molecular recognition and biosensing. Biomimetics 5(2), 20.

Schultz, J.S. (1982) Optical sensor of plasma constituents, Google Patents.

Sedki, M., Chen, X., Chen, C., Ge, X., Mulchandani, A., 2020. Non-lytic M13 phage-based highly sensitive impedimetric cytosensor for detection of coliforms. Biosens Bioelectron 148, 111794.

Suzuki, S., Takahashi, F., Satoh, I., Sonobe, N., 1975. Ethanol and lactic acid sensors using electrodes coated with dehydrogenase—Collagen membranes. Bulletin of the Chemical Society of Japan 48(11), 3246-3249.

Svetlicic, E., Jaén-Luchoro, D., Klobucar, R. S., Jers, C., Kazazic, S., Franjevic, D., et al. (2023). Genomic characterization and assessment of pathogenic potential of Legionella spp. isolates from environmental monitoring. Front. Microbiol. 13:1091964. doi: 10.3389/fmicb.2022.1091964

Svetlicic, E., Jaén-Luchoro, D., Klobucar, R.S., Jers, C., Kazazic, S., Franjevic, D., Klobucar, G., Shelton, B.G., Mijakovic, I., 2023. Genomic characterization and assessment of pathogenic potential of Legionella spp. isolates from environmental monitoring. Front Microbiol 13:1091964. https://doi.org/10.3389/fmicb.2022.1091964

Vadde, K.K., Feng, Q., Wang, J., McCarthy, A.J., Sekar, R., 2019. Next-generation sequencing reveals fecal contamination and potentially pathogenic bacteria in a major inflow river of Taihu Lake. Environ Pollut 254. <https://doi.org/10.1016/j.envpol.2019.113108>

Vestergaard, M.d.C., Tamiya, E., 2015. Nanobiosensors and nanobioanalyses: A review. J Nanobiosens Nanobioanalyses, 3-20.

Vierheilig, J., Savio, D., Farnleitner, A. H., Reischer, G. H., Ley, R. E., Mach, R. L., et al. (2015). Potential applications of next generation DNA sequencing of 16S rRNA gene amplicons in microbial water quality monitoring. *Water Sci. Technol.* 72, 1962–1972. doi: 10.2166/wst.2015.407

Yoo, E.-H. and Lee, S.-Y., 2010. Glucose biosensors: an overview of use in clinical practice. Sensors (Basel) 10(5), 4558-4576.
